# Rapid Access to Small Molecule Conformational Ensembles in Organic Solvents Enabled by Graph Neural Network-Based Implicit Solvent Model

**DOI:** 10.1021/jacs.4c17622

**Published:** 2025-04-10

**Authors:** Paul Katzberger, Lea M. Hauswirth, Antonia S. Kuhn, Gregory A. Landrum, Sereina Riniker

**Affiliations:** Department of Chemistry and Applied Biosciences, ETH Zürich, Vladimir-Prelog-Weg 2, Zürich 8093, Switzerland

## Abstract

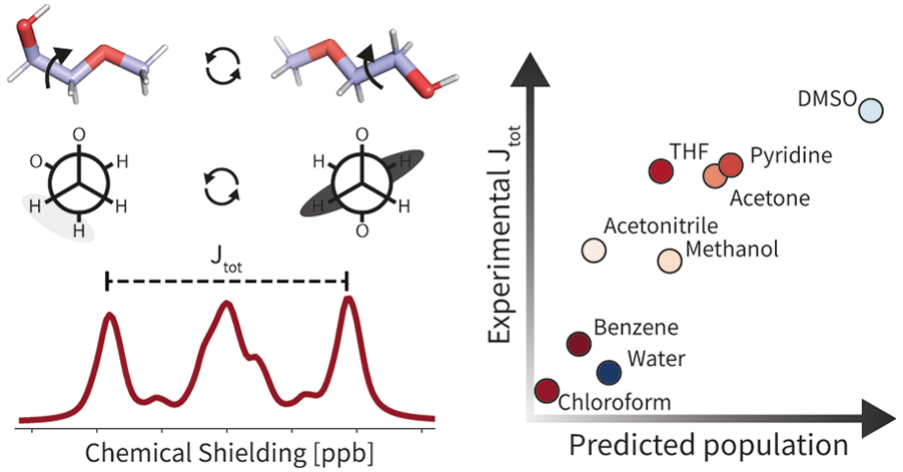

Understanding and manipulating the conformational behavior of a molecule in different solvent environments is of great interest in the fields of drug discovery and organic synthesis. Molecular dynamics (MD) simulations with solvent molecules explicitly present are the gold standard to compute such conformational ensembles (within the accuracy of the underlying force field), complementing experimental findings and supporting their interpretation. However, conventional methods often face challenges related to computational cost (explicit solvent) or accuracy (implicit solvent). Here, we showcase how our graph neural network (GNN)-based implicit solvent (GNNIS) approach can be used to rapidly compute small molecule conformational ensembles in 39 common organic solvents reproducing explicit-solvent simulations with high accuracy. We validate this approach using nuclear magnetic resonance (NMR) measurements, thus identifying the conformers contributing most to the experimental observable. The method allows the time required to accurately predict conformational ensembles to be reduced from days to minutes while achieving results within one *k*_B_*T* of the experimental values.

## Introduction

The majority of marketed pharmaceuticals are low-molecular-weight organic compounds, commonly referred to as small molecules.^1^ In solution, flexible small molecules are dynamic entities, giving rise to a Boltzmann-weighted ensemble of different conformations, which depends on the nature of the surrounding solvent. Weak interactions can vary dramatically between different solvents, resulting in critical differences between the corresponding conformational ensembles.^2,3^ Therefore, understanding even slight variations in the populations of conformers is crucial for a variety of applications. These include the synthesis of regio- and stereospecific molecules,^4,5^ optimization and understanding of passive membrane permeability,^6,7^ as well as fine-tuning physical properties.^8^ In addition, differing conformational ensembles also give rise to distinct experimental observables, and thus, they strongly influence the interpretation of experimental techniques. For instance, nuclear magnetic resonance (NMR),^9−12^ infrared (IR) spectroscopy,^13,14^ or vibrational circular dichroism (VCD) spectroscopy^15−19^ among others exhibit all conformational dependencies.

While molecular dynamics (MD) simulations with explicit solvent molecules (and if needed enhanced sampling techniques) constitute a powerful tool to estimate the conformational ensemble of small molecules in solution,^20−23^ they are inherently constrained by their relatively high computational demand. The molecule of interest (the solute) is solvated in a large box of solvent molecules, and the whole system is propagated through time. This approach is inherently inefficient since the probabilities of the conformational ensemble are identified from their repeated occurrence in the simulation rather than their intrinsic free energy, which is not readily accessible from explicit-solvent simulations. Nevertheless, such simulations are currently the gold standard because implicit solvation methods (e.g., Poisson–Boltzmann (PB),^24^ fast analytical continuum treatments of solvation (FACTS),^25^ or generalized Born (GB) models^26^) do not achieve the desired accuracy.

Machine learning has been proposed to bridge this gap^27−32^ and in previous studies,^33,34^ we have shown for the case of water that a graph neural network (GNN) trained on reference forces from explicit-solvent simulations can represent the solvent in a probabilistic way and, hence, accurately simulate a system using many fewer degrees of freedom. This reduces the computational effort significantly while providing comparable results to explicit-solvent simulations. The approach is inspired by the concept of Δ-learning^35^ and incorporates the functional form of the traditional GB-SA model as a physics-based regularization^36^ to reproduce the reference forces. This ensures the correct physical behavior of the model.

Here, we extend the GNN-based implicit solvent (GNNIS) model to 39 common organic solvents and demonstrate how the approach can be employed to rapidly identify Boltzmann-weighted conformational ensembles of small molecules in these solvents. To encode the different solvents in the GNN, whose architecture is described in greater detail in our previous work,^33,34^ we use an embedding scheme that allows the model to learn itself how to represent each solvent. Our approach allows for sets of randomly sampled conformations (e.g., from a conformer generator) to be minimized into Boltzmann-weighted conformational ensembles that are on par with explicit-solvent simulations. The approach is tested using ∼200 experimental observables derived from new NMR measurements as well as literature data. These tests show high accuracy using minimal computational effort and predict solvent-specific differences within one *k*_B_*T* of experimental results. This high speed (e.g., a small molecule’s conformational ensemble can be obtained in minutes rather than days) will allow the study of large data sets as well as iterative design procedures.

## Results and Discussion

### Technical Validation

**Figure 1:**
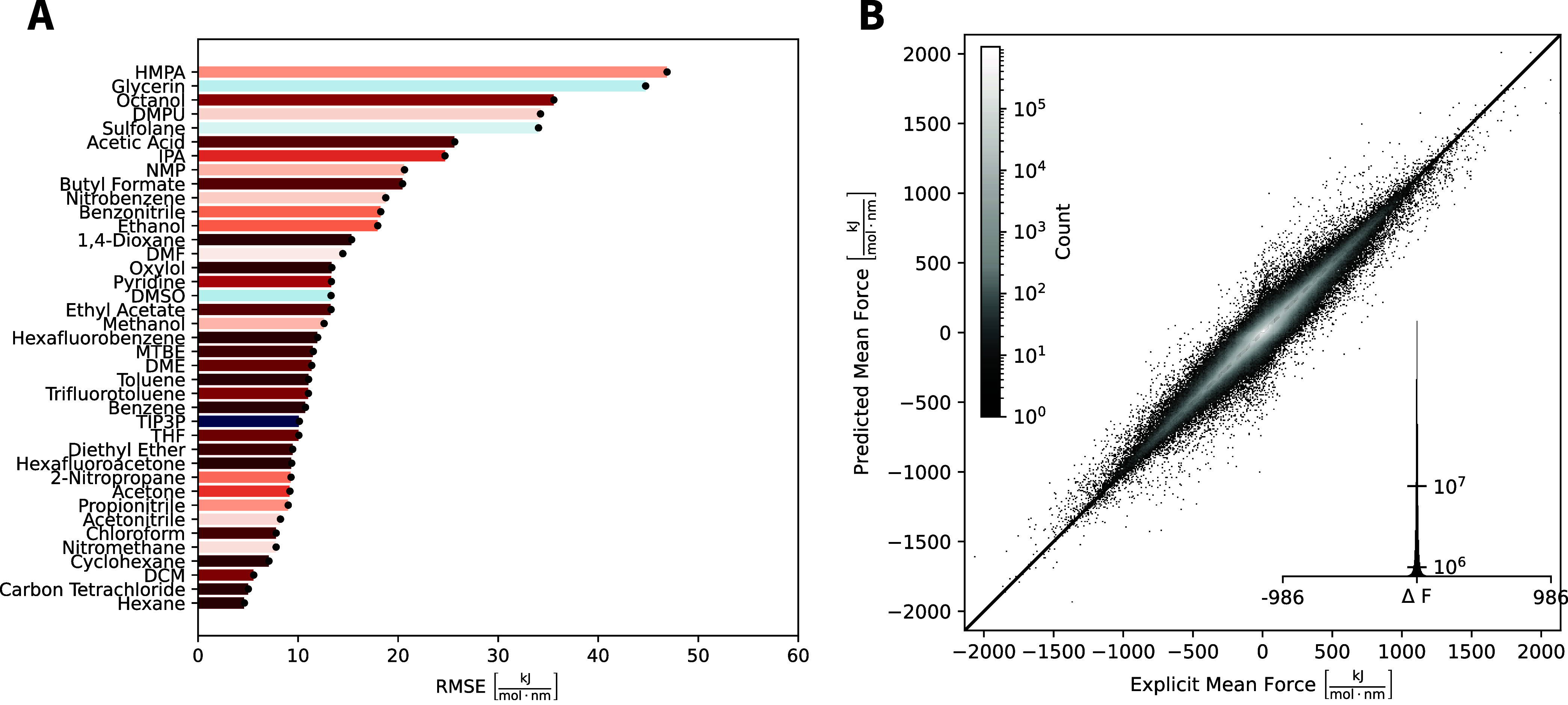
Comparison of the GNN predicted versus reference forces. (A) Root-mean-square error (RMSE) for the 39 solvents. The black error bar indicates the standard deviation among the three GNN models trained with different random seeds. The color scale corresponds to the dielectric permittivity of the solvent. (B) Correlation between the predicted and reference forces for one replicate of the GNN.

The accuracy of the GNN, trained on ∼370,000 diverse small molecules with a molecular weight <500 Da, was assessed on an external test set consisting of 1000 compounds with a molecular weight ranging from 500 to 700 Da. The RMSE values achieved for each solvent are shown in Figure 1A. The overall correlation between the GNN and the external test set is excellent (Figure 1B). The deviation between predicted and reference forces varies significantly between different solvents ranging from 4.6 kJ mol^–1^ nm^–1^ for hexane to 46.9 kJ mol^–1^ nm^–1^ for HMPA. These differences between the solvents are most likely an effect of differing force magnitudes (see Supporting Information Figure S5 for the distribution of the magnitude of reference forces). Apolar solvents generally feature smaller forces, while polar ones feature larger forces. In addition to these polarity effects, other aspects are likely the influence of the conformational flexibility, self-diffusion, and rotational correlation time of the solvent (i.e., the time it takes for a solvent to equilibrate around a given conformation), which could explain why HMPA, glycerin, and octanol show the largest errors. While for most solvents, the chosen sampling time to capture the Boltzmann-weighted ensemble of solvent configurations around a given conformer was long enough, these larger solvents may suffer from increased sampling errors in the test-set calculations. However, as the GNN was trained on multiple conformers, it is possible that the lower test-set accuracy does not manifest in large deviations in prospective applications (e.g., free-energy profiles or conformational ensembles). For this reason, the sampling time for the larger solvents was not increased when generating the training set.

Three models with different random seeds for the initialization were trained and evaluated. In general, the performance of the three models was virtually identical (i.e., standard deviation over the RMSE values is at most 0.05 kJ mol^–1^ nm^–1^). Hence, one model was selected for all further analysis. The model with the highest average RMSE for all solvents was chosen to be conservative.

### Solvent Embedding

**Figure 2:**
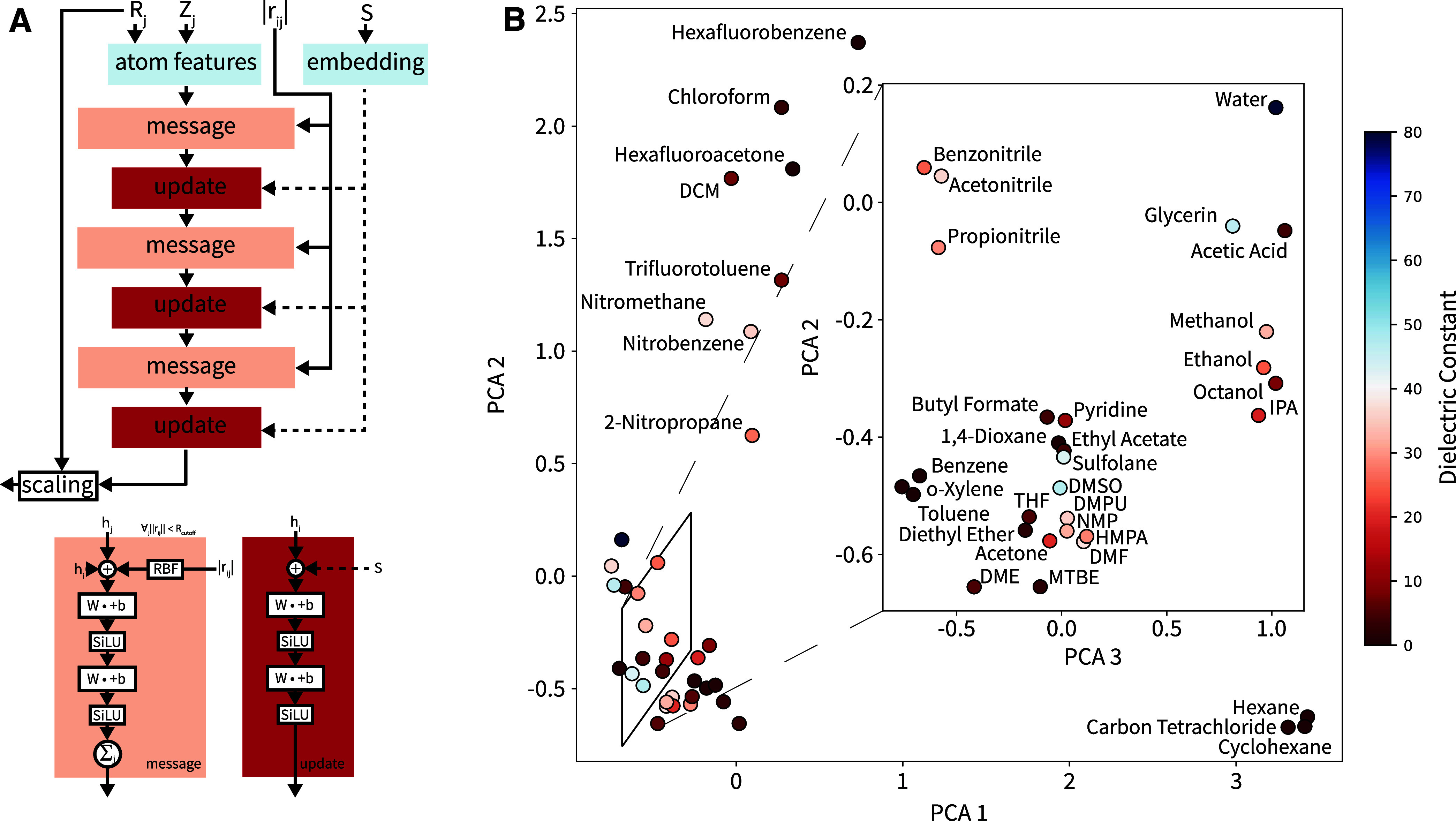
(A) Schematic representation of the GNN architecture used for the implicit solvent. Message-passing steps are shown in coral, while node-wise operations are shown in red. (B) Principal component analysis (PCA) of the solvent embedding. The first two PCs are shown in the full plot. The inset further resolves the closely related solvents in the lower left corner by showing the projection with respect to the second and third PCs. The color scale corresponds to the dielectric permittivity of the solvent.

A key aspect of our GNN architecture (Figure 2A) is the simultaneous training of the entire solvent set. Briefly, a single model is trained for the entire set of solvents, and differences among solvents are captured through the embedding of solvent-specific feature vectors. This concurrent training should optimize information gain and efficiency by allowing the GNN to exploit transfer learning among solvents, i.e., to link similar solvent behavior to related features. While this means that the model needs to be retrained, if a new solvent is added, the cost of training a new model is negligible compared to the cost of generating a training set for a new solvent. To probe the GNN’s ability to develop an artificial “chemical intuition”, the feature vectors of the 39 solvents were taken from the solvent embedding layer of the GNN, and a principal component analysis (PCA) was performed on them. In Figure 2B, the three largest principal components, which together account for 47% of the variance, are shown. The resulting 3D arrangement of solvents groups chemically similar solvents (e.g., having the same functional group) together, in line with a chemist’s intuition and going beyond a solvent classification based solely on dielectric permittivity. For example, THF and DMSO have very different dielectric permittivities but still appear close in the PCA projection as they are both polar aprotic solvents. On the other hand, solvents with similar dielectric permittivity but different functional groups are far apart in our projection (e.g., chloroform and acetic acid). This demonstrates that the GNNIS model does indeed identify solvent effects that are not accounted for in classical continuum-based implicit solvent models and supports our strategy to train multiple solvents in a joint manner.

### Prospective Molecular Dynamics Simulations

**Figure 3:**
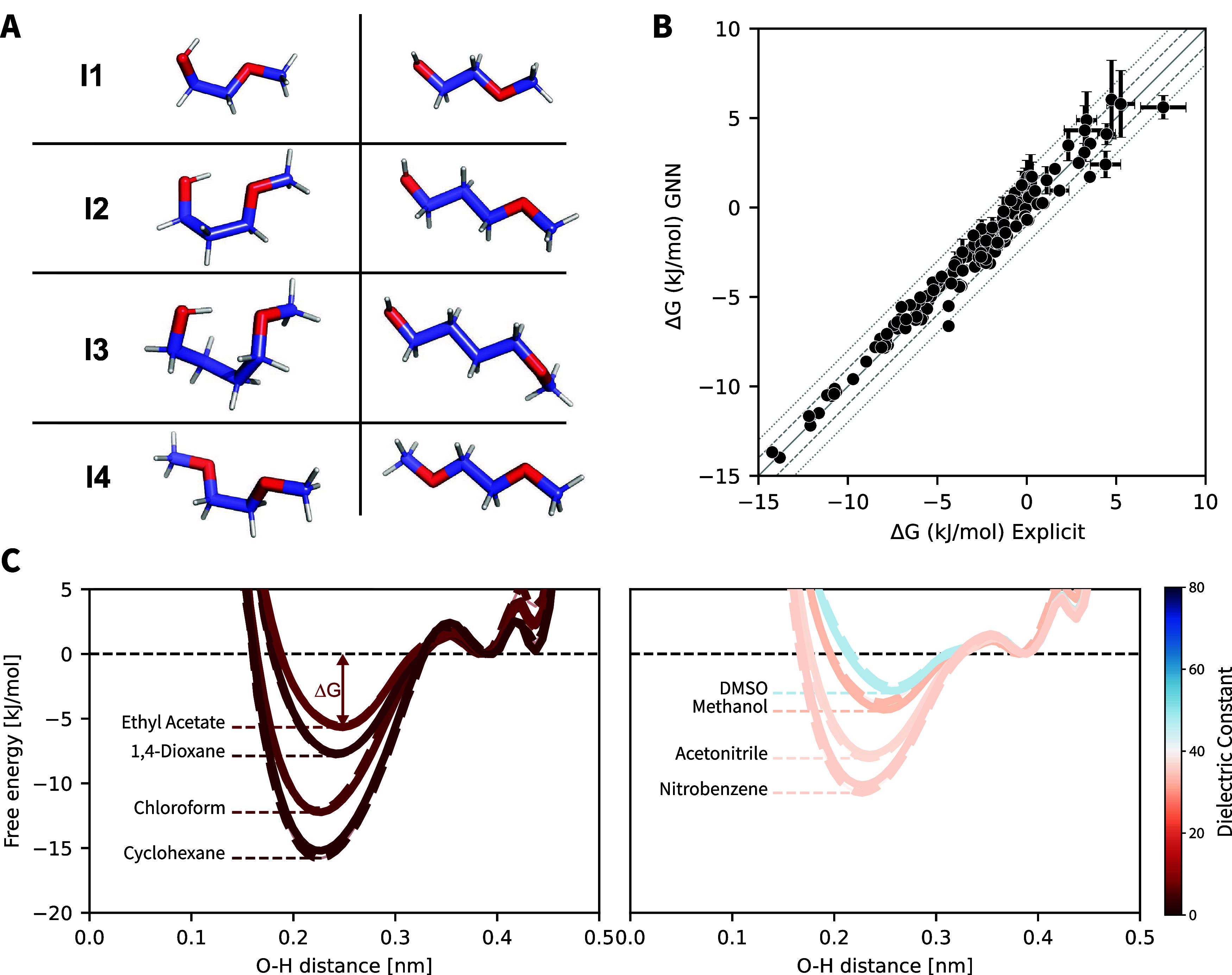
Comparison of prospective MD simulations using the GNNIS model (10 × 50 ns) compared to the explicit-solvent reference (3 × 500 ns). (A) Compound set I with closed (left) and open (right) conformations. (B) Comparison of the free-energy difference Δ*G* between the lowest lying minima of the closed and opened intramolecular hydrogen bond for the explicit-solvent simulations (*x*-axis) and the GNNIS simulations (*y*-axis). Each dot represents one molecule–solvent combination. Error bars indicate the standard deviation across the simulation replicates. Error bars below 0.5 kJ mol^–1^ are omitted for clarity. (C) Free-energy profiles of the formation of the intramolecular hydrogen bond of compound I1 simulated using explicit solvent (dashed lines) and the GNNIS model (solid lines). Low dielectric solvents are shown on the left: ethyl acetate, 1,4-dioxane, chloroform, and cyclohexane; high dielectric solvents on the right: DMSO, methanol, acetonitrile, and nitrobenzene. The color scale indicates the dielectric permittivity of the solvent. The free-energy difference Δ*G* between the global minimum and the lowest-lying local minimum for ethyl acetate is indicated by the red double arrow.

Next, we probed the ability of the GNNIS model to reproduce explicit-solvent MD simulations on an additional compound set I (Figure 3A) whose members were not part of the training set. Crucially, all compounds in set I can form intramolecular as well as solvent-intermediated hydrogen bonds and should thus be susceptible to solvent effects that are not simply correlated with the dielectric permittivity of the solvent. The free-energy profiles of the intramolecular hydrogen bonds (Supporting Information Figures S6–S9) and the free-energy differences Δ*G* between the conformations with the open and closed hydrogen bond were extracted from both the GNNIS and explicit-solvent MD simulations. Given that the free-energy difference is obtained from populations, note that the persistence time, simulation length, and write-out frequency have an effect on the range of possibly detectable free-energy differences. To ensure that the compared results do not lie outside of this range, the variability of the results was analyzed by splitting the explicit-solvent reference simulation into three equal parts and analyzing ten independent replicates for the GNNIS simulations. The comparison shown in Figure 3B demonstrates the excellent agreement between the approaches.

The complete free-energy profiles of compound I1 in a selection of four low dielectric solvents (i.e., dielectric permittivity ϵ < 10) and four high dielectric solvents (i.e., ϵ > 30) are depicted in Figure 3C. As expected, the profiles in the individual solvents diverge significantly despite their similar dielectric permittivities, which can be reproduced by the GNNIS model. In contrast, simulations with the state-of-the-art implicit solvent model GB-Neck2^37^ show significant deviations from the explicit-solvent reference. The median absolute error with GB-Neck2 is 2.5 kJ mol^–1^ compared to 0.6 kJ mol^–1^ for GNNIS (Supporting Information Figures S10–S14). Note that the GB-Neck2 model was optimized to best reproduce simulations in water and not in other solvents. To ensure that the observed limitations are not an artifact of this optimization, a second GB-based model, the GB-OBC model,^38^ was also compared, leading to qualitatively equal results (see Supporting Information Section S4). For this reason, and because the GNNIS model is based on the GB-Neck2 model, the further comparisons are only performed with the GB-Neck2 model.

The high accuracy of the GNNIS model is especially significant as it is achieved at a substantially reduced cost compared to explicit-solvent simulations when multiple systems are simulated in parallel (as in this study). While an explicit-solvent simulation can make full use of currently available hardware (i.e., the GPU), a single GNNIS simulation does not make full use of it and allows the evaluation of multiple systems in parallel, achieving a speed-up of 10-fold (1750 versus 16900 ns d^–1^) on the same hardware (8 CPU cores + 1 NVIDIA RTX 4090 GPU). A detailed description of the parallelization approach and the relationship between simulation speed and system size are given in the Supporting Information Sections S5 and S6.

### Rapid Assessment of Conformational Ensembles

A key advantage of implicit-solvent methods is that they allow for the direct estimation of a conformer’s solvation free energy.^39^ In contrast, with explicit-solvent simulations, these computations require costly free-energy calculations. Implicit-solvent methods can hence be used to identify minima of a conformational ensemble and to calculate the corresponding free energies. This enables rapid access to Boltzmann-weighted conformational ensembles.

**Figure 4:**
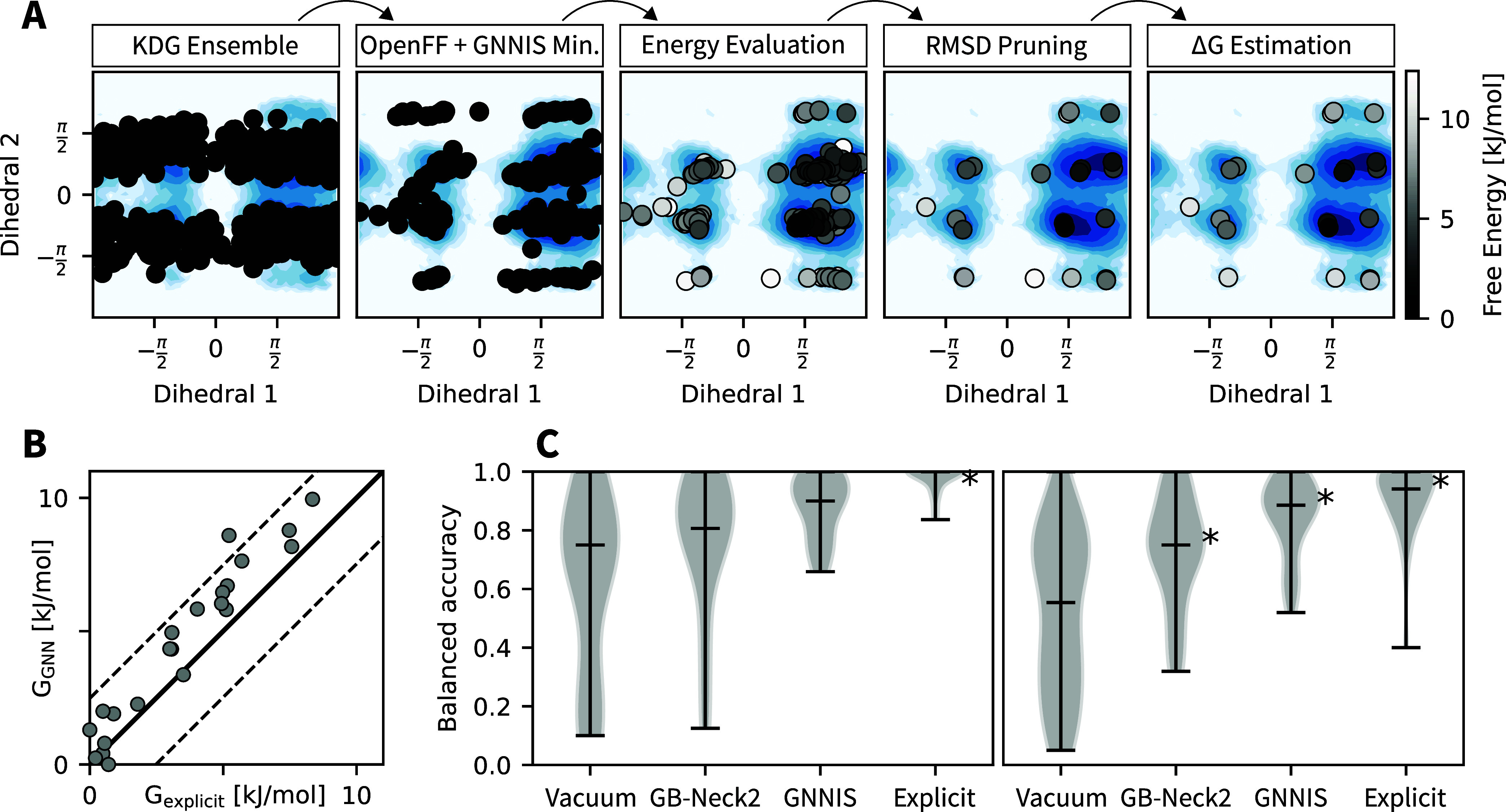
(A) Example of the minimization workflow for 512 conformers of compound C1 in water. Values of the two dihedral angles around the amide bond of C1 are represented as black dots for the various conformers. The reference free-energy profile is depicted by the blue heatmap. From left to right, the steps in the workflow include: (1) generating random conformers (here with KDG^42^), (ii) minimizing these conformers using OpenFF^43^ for the intramolecular interactions combined with the GNNIS model, (iii) sorting conformers by their potential energy, (iv) RMSD pruning of the ensemble, and (v) estimating free energies. (B) Comparison of the estimated free energy of the final set of conformers with the free energy computed from the explicit-solvent reference simulation of C1 in water. Dashed lines indicate a difference of 1 *k*_B_*T* from the identity line. (C) Comparison of the conformer ensembles minimized in vacuum, using GB-Neck2, or using the GNNIS model for compound set C (left) and P (right). Models are compared based on the balanced accuracy score of finding conformers below 1 *k*_B_*T*. The sampling error of the explicit-solvent simulation is given for reference. Statistically significant differences (*p* < 0.05) between a model and the model to its left are indicated by a star. The median balanced accuracy is indicated by the horizontal line.

The accuracy of conformational ensembles generated with conventional implicit solvent models has been limited by their deficiencies in the description of explicit solvent effects (e.g., relative stability of intramolecular hydrogen bonds, see Supporting Information Figures S10–S14). On the other hand, the GNNIS model does, as shown above, accurately reproduce such explicit solvent effects. We further explored this approach for rapid identification of Boltzmann-weighted conformational ensembles of small molecules. The procedure used is depicted in Figure 4A (see Methods Section for more details). Briefly, a diverse set of conformers is generated randomly using distance geometry^40−42^ and minimized using a standard force field (OpenFF 2.0.0^43^) in combination with the GNNIS model. The resulting ensemble is sorted based on the potential energy of each conformer and pruned based on the root-mean-square distance (RMSD). In the last step, the free energies of the final conformers are estimated based on the predicted energies and an entropy estimation using Grimme’s quasi-RRHO approach,^44^ whereby the entropy of all modes is damped and complemented with a free-rotor entropy contribution.

#### Simulated Ensembles

For two compound sets C and P (see Figure 8 in the Methods section), which feature smaller (i.e., molecular weight <500 Da) and larger molecules (i.e., molecular weight >500 Da), respectively, the GNNIS-predicted ensembles were compared to ensembles extracted from explicit-solvent simulations. As shown in Figure 4B for one of the compounds in water, the GNNIS-predicted ensembles are generally in good agreement with the explicit-solvent reference. The results for all other solvent–compound combinations are provided in Supporting Information Figures S15 and S19 for sets C and P, respectively, for the GNNIS model and in Supporting Information Figures S16–S18 and S20–S22 for the other solvent models.

To quantitatively assess the quality of the conformational ensemble, we calculated balanced accuracies for ensembles minimized using OpenFF 2.0.0 with (i) no solvent (in vacuum), (ii) the GB-Neck2 implicit solvent model, and (iii) the GNNIS model, and for ensembles simulated with the same force field in explicit solvent. The balanced accuracy indicates whether low-lying minima (i.e., minima with free energies below one *k*_B_*T*) are correctly identified in an ensemble. An explanatory illustration is provided in Supporting Information Figure S23. The correct prediction of low-energy conformers is especially important as they contribute significantly to experimental observables, e.g., NMR or IR spectra. As shown in Figure 4C, the GNNIS minimization detects low-lying free-energy minima with high accuracy, significantly outperforming GB-Neck2. In the case of set P, the minimization procedure with the GNNIS model even approaches the balanced accuracy obtained with explicit-solvent simulations evaluated using standard sampling times (i.e., 50 and 100 ns REST2 simulations for set C and P, respectively). As the compounds of set P are larger and more flexible compared to those in set C, longer simulation times in explicit solvent would be required to further increase the achievable balanced accuracy.

These results are very promising as the computational cost of the proposed workflow is much lower than that of explicit solvent simulations. The entire pipeline depicted in Figure 4A took minutes rather than hours for the reference calculations. A detailed analysis of the computational cost of the approach and the reference simulations is provided in the Supporting Information Section S7.

#### Comparison with Experimental Observables

**Figure 5:**
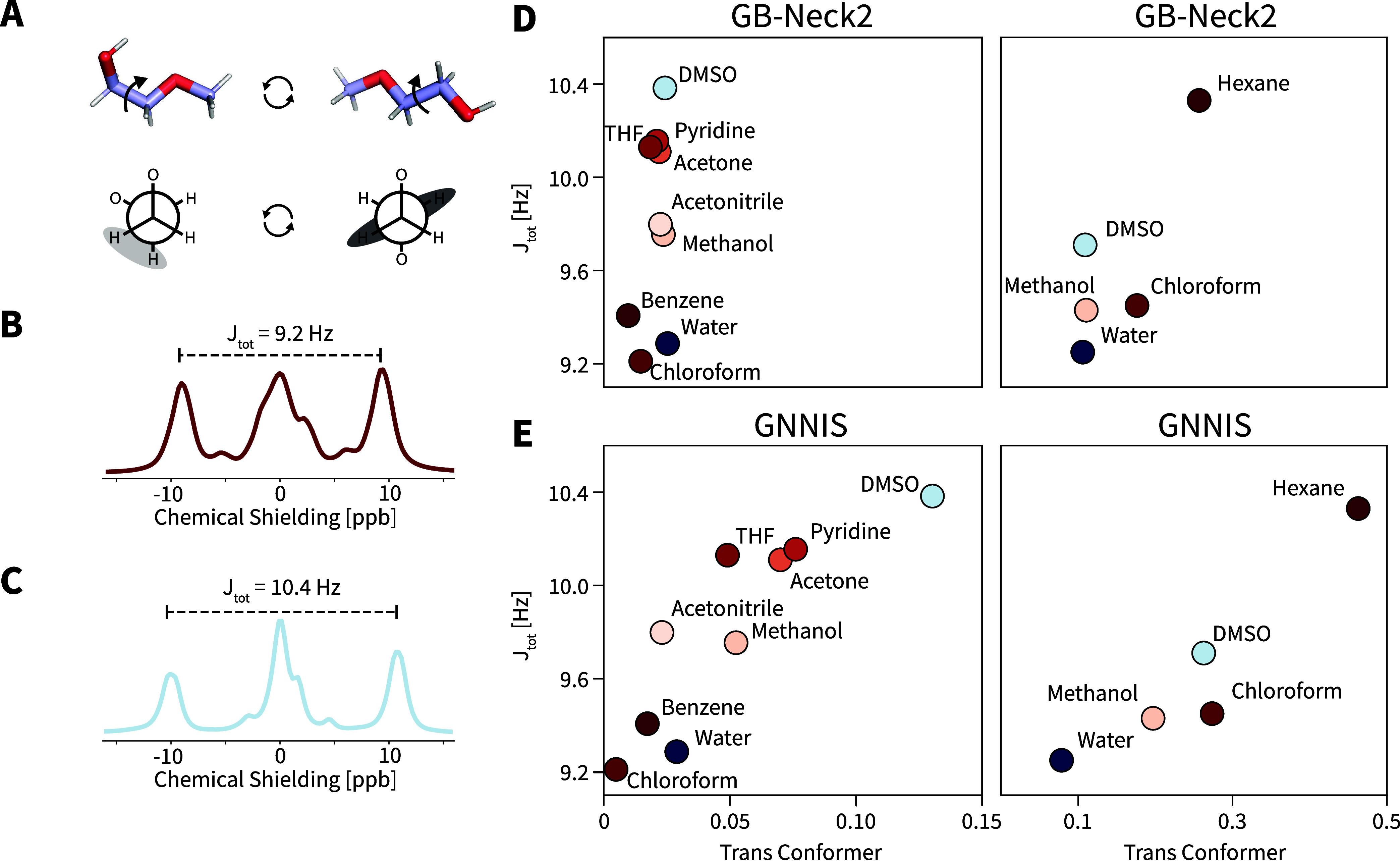
Assessment of the quality of the ensembles minimized with the classical GB-Neck2 or GNNIS model. (A) Schematic representation of the interconversion of the two key conformations (top) and Newman projections of the gauche and trans conformation (bottom) of compound I1. (B) ^1^H NMR spectrum of the characteristic H_α_ proton peak used for the determination of *J*_tot_ in chloroform. (C) ^1^H NMR spectrum of the characteristic H_α_ proton peak used for the determination of *J*_tot_ in DMSO. (D) Comparison of the predicted population of the trans conformer (*x*-axis) and the experimental J_tot_ coupling constant (*y*-axis) of I1 (left) and I4 (right, experimental data taken from ref (45).) for the GB-Neck2 model. (E) Same comparison for the GNNIS model.

To further explore the applicability of the conformational ensembles minimized with the GNNIS model for practical applications, we followed the workflow described above for compounds I1 and I4 (Figure 3A) in solvents for which proton NMR measurements with high spectral resolution are available. Experimental measurements for I1 were conducted in parallel to the computational work, while data for I4 was taken from the literature.^45^ At room temperature, both I1 and I4 feature two dominant conformers that interconvert (Figure 5A). The relative population of the conformers can be directly deduced from the *J*-coupling constant of the α-protons of the methoxy-group (denoted H_α_ in the following). The NMR signal of H_α_ is a multiplet resulting from two distinct vicinal proton–proton *J*-couplings (*J*_A–B_, *J*_A–C_). Both *J*_A–B_ and *J*_A–C_ are rotational averages over all conformers. Here, we extracted the total scalar coupling *J*_tot_ = *J*_A–B_ + *J*_A–C_ as the frequency difference between the two outermost peaks of the multiplet. In chloroform, the predominant population of the gauche conformer minimizes *J*_tot_ (Figure 5B), while a higher value is observed in DMSO from an enhanced population of the trans-conformer (Figure 5C).

The correlation between the populations of the trans-conformer for the predicted ensemble and the experimental observable for compounds I1 and I4 is shown in Figure 5D,E for GB-Neck2 and GNNIS, respectively. For both compounds, the expected linear relationship between the predicted trans-conformer population and *J*_tot_ is observed for the ensembles minimized with OpenFF 2.0.0 and GNNIS. The results indicate that the proposed workflow can rapidly provide solution ensembles and represents a significant improvement over the GB-Neck2 model. One interesting aspect of these results is that the relative populations of the opened and closed conformations of compound I1 do not align with the perception that polar solvents should favor more open conformations, as can be seen in the GB-Neck2 results. As the GB-Neck2 model relies solely on a continuum description using a fixed dielectric constant, the predicted populations are dependent on this property. Water, DMSO, and methanol, which feature the highest dielectric permittivities, are predicted to have the greatest fraction of the trans-conformer (conversely, benzene, with the lowest dielectric permittivity, is predicted to have the smallest fraction of the trans-conformer). While this is true for DMSO, water features one of the lowest populations of the trans-conformer for both compounds I1 and I4. Further investigation revealed that the reason for this is the ability of water to form an hydrogen-bonding network with the compound (see Supporting Information Figure S24), thus stabilizing the closed conformation. The fact that the GNNIS model can capture the effects of these complex interactions is especially promising.

The two main outliers are acetonitrile and THF for compound I1 (left panel of Figure 5E) for which the prediction diverges from the linear trend. However, the explicit-solvent simulations of compound I1 in these solvents (see Section “Prospective Simulations”) indicate that this deviation may not be due to GNNIS itself but rather to the explicit-solvent model that was used to generate the training data. The predicted fraction of the trans-conformer based on the explicit-solvent simulation (2.9% and 6.0% for acetonitrile and THF, respectively) is very similar to that predicted by the minimization approach with OpenFF 2.0.0 and GNNIS (2.3% and 4.9% for acetonitrile and THF, respectively). Note that while a divergence to experiment could also arise from issues of the underlying solute force field, these differences should be systematic for all solvents and result in a shift of all populations rather than manifest in a single outlier for a specific solvent.

**Figure 6:**
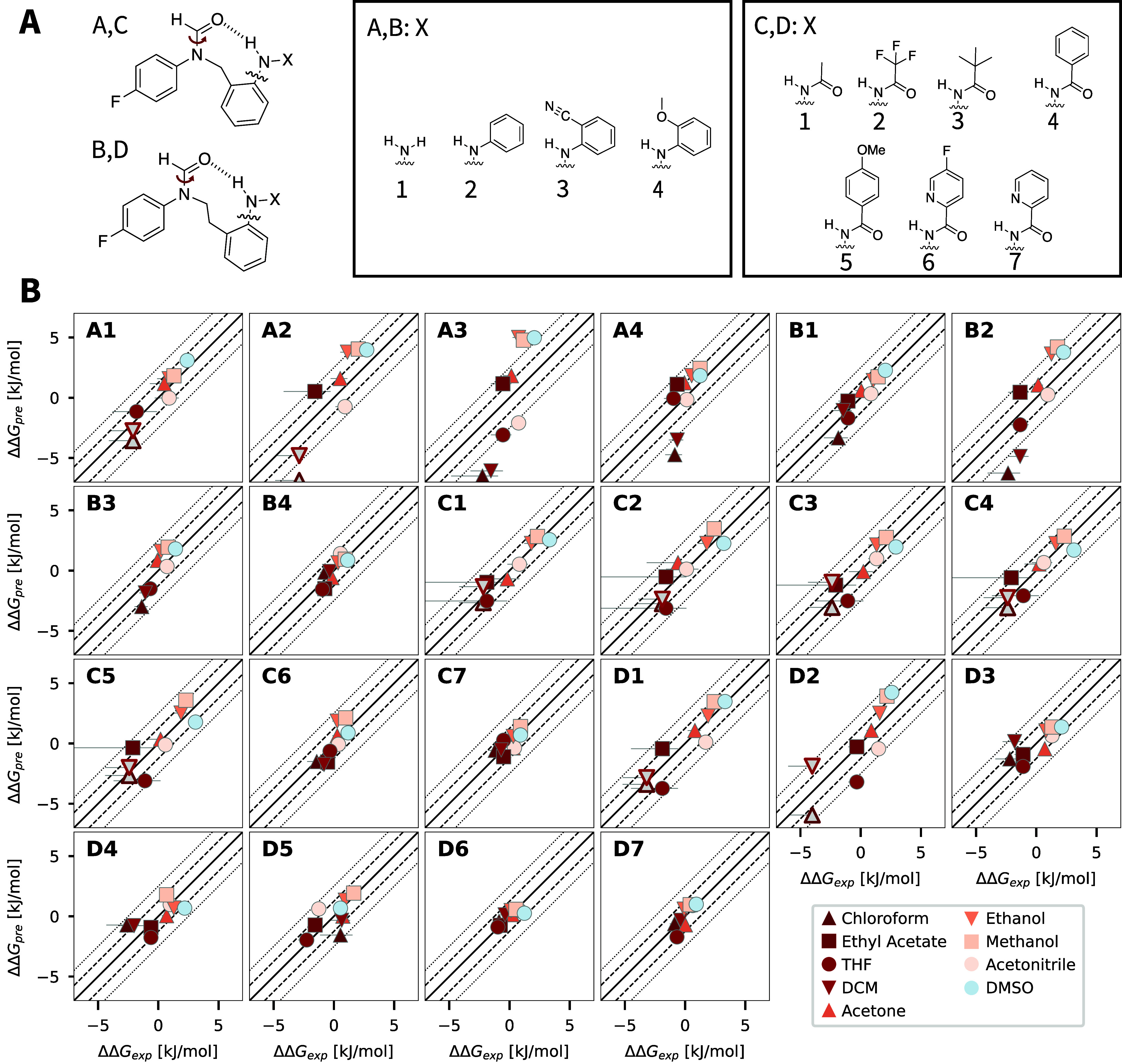
(A) Illustration of the 22 studied molecular balances. (B) Comparison of predicted ΔΔ*G* values using the GNNIS model and the experimental data for the 22 molecular balances in nine different solvents. The color scale indicates the dielectric permittivity of the solvent. The experimentally determined standard error is denoted by the black error bars. Markers filled with gray represent measurements where the experiment could not determine the free-energy difference between the two rotations exactly. In these cases, the value indicated for these points provides an upper limit to the undefined values.

In order to further investigate the behavior of the proposed minimization workflow, a set of 22 molecular balances (Figure 6A) designed to quantify hydrogen-bond strength in different solvents was studied. For these compounds, Meredith et al.^46^ measured the distribution of two distinct ketone rotations (Figure 6A) by means of ^19^F{^1^H} NMR measurements in nine different solvents. These rotations give strong evidence of the strength of the intramolecular hydrogen bond and the composition of the conformational ensemble of the molecule. The computational prediction of such properties would be highly desirable in fields such as synthetic organic chemistry, and makes them an ideal test case for the GNNIS model.^46^

We performed our minimization approach for all balances in the different solvents. The main question we wanted to address in this context is how effectively the minimized ensembles can capture small differences in the populations of the different rotational states in varying solvent environments. To compare these, we first used the minimized ensemble to predict the free-energy difference between the two ketone rotation states. Next, the difference between these predictions and their mean (ΔΔG_pre_) is compared to the experimentally determined free-energy differences and their mean (ΔΔG_exp_). The results are shown in Figure 6B. Overall, the GNNIS model reproduces the differences with respect to the conformational ensembles in the different solvents very accurately, with most of the ΔΔ*G* values within one *k*_B_*T*. This is especially interesting as this rotation, based on experimental findings, takes place on the μs time scale, making the analysis with explicit-solvent simulations computationally expensive. Comparing the results to GB-Neck2 simulations highlights the improvement over state-of-the-art conventional implicit solvent methods (see Supporting Information Figure S25 and S26). Ensembles minimized with GB-Neck2 predict almost the same values for the different polar solvents and the strength of the solvent effect is significantly underestimated (i.e., the median slope is 0.43). The high accuracy and low computational effort suggest that the GNNIS model is well-suited to study these kinds of systems.

## Conclusions

This study extends the GNNIS approach to 39 organic solvents and presents a workflow for rapidly identifying Boltzmann-weighted conformational ensembles of small molecules in solution in combination with standard force fields. The GNNIS model was trained on a diverse set of small molecules simulated in the 39 different solvents, validated based on prospective MD simulations, and tested on conformational preferences determined from NMR experiments. Both comparisons show excellent agreement of the method and the reference data. Further, the dramatic speed-up compared to explicit-solvent simulations (i.e., ensembles can be generated within minutes rather than hours) makes the proposed approach a powerful tool for understanding the behavior of molecules in solution. This advance could facilitate the rational design of molecules by enabling the study of larger data sets and faster turnarounds, as well as the accurate and rapid interpretation of experimental observables. The software is provided open-source, and the training data is made freely available.

## Methods

### Solvents

39 solvents relevant for organic synthesis were selected (ordered by descending dielectric permittivity): Water, dimethyl sulfoxide (DMSO), glycerin, sulfolane, dimethylformamide (DMF), nitromethane, acetonitrile, *N*,*N*′-dimethylpropyleneurea (DMPU), nitrobenzene, methanol, *N*-methylpyrrolidone (NMP), propionitrile, hexamethylphosphoramide (HMPA), 2-nitropropane, benzonitrile, ethanol, acetone, isopropyl alcohol (IPA), pyridine, octanol, trifluorotoluene, dichloromethane (DCM), tetrahydrofuran (THF), dimethyl ether (DME), ethyl acetate, acetic acid, butyl formate, chloroform, methyl tert-butyl ether (MTBE), diethyl ether, oxylol, toluene, benzene, carbon tetrachloride, 1,4-dioxane, hexafluoroacetone, hexafluorobenzene, cyclohexane, hexane. The density and dielectric permittivity values of the solvents were taken from the literature^47−52^ and are given in Supporting Information Table S1.

### Generation of the Training and Test Sets

The training and external test sets for water were taken from ref (34). The same procedure was followed for the calculation of the additional solvents but with fewer conformers per molecule (i.e., nine conformers were used for water): three conformers for chloroform, DMSO, and methanol, and one conformer for the remaining solvents. As in ref (34), forces could not be extracted for all compound–solvent combinations as some simulations were unstable and led to infinite energies. These combinations were not added to the data set. The final data set is freely available in the ETH Research Collection (10.3929/ethz-b-000710355).

### Graph Neural Network (GNN)

The GNN architecture is shown in Figure 2A. It is based on our previous work in ref (34) but extends the approach for the prediction of energies and forces in multiple solvents. The model represents an invariant GNN with three passes. The additional solvent embedding was implemented using a lookup table that carries a vector of length 64 with learnable weights for each solvent. These weights were optimized during training together with all other trainable parameters. A detailed description of the architecture is given in the Supporting Information Section S1 “Graph Neural Network”.

For the training process, 95% of the full data set was used while the remaining 5% was reserved for validation. The training was carried out over 50 epochs with a batch size of 256. An exponentially decaying learning rate was applied, ranging from 5 × 10^–4^ to 5 × 10^–6^. The mean squared error was selected as the loss function, and the Adam optimizer^53^ was employed for weight optimization, with gradients being clipped to a norm of 1. Additionally, a dropout value of 0.1 was employed during training.

### Molecular Dynamics (MD) Simulations

The MD simulations were carried out using the methodology described in our previous work.^34^ The ETKDG^42^ conformer generator as implemented in the RDKit^54^ was used to generate the starting structures. Molecules were parametrized using the OpenFF 2.0.0 force field^43^ and simulated using the OpenMM (version 8.0.0) simulation program.^55^ The solvation of compounds was performed using the PACKMOL program^56^ with a padding of 1 nm. The L-BFGS algorithm was used to minimize all compounds with the tolerance set to 10 kJ mol^–1^ nm^–1^. During simulation, all bonds involving hydrogens were constrained using the SETTLE^57^ for water and CCMA^58^ algorithms for all other bonds. Langevin dynamics employing the LFMiddle discretization scheme^59^ was used with a Monte Carlo barostat to propagate the system. The reference temperature and pressure were set to 300 K and 1 bar, respectively. Nonbonded interactions were corrected using the particle mesh Ewald scheme^60^ with a nonbonded cutoff of 1 nm. The time step of all simulations was set to 2 fs. The center-of-mass motion was removed using OpenMM’s CMMotionRemover, and the write-out frequency was set to 1000 and 100 for simulations using explicit and implicit solvent models, respectively.

**Figure 7:**
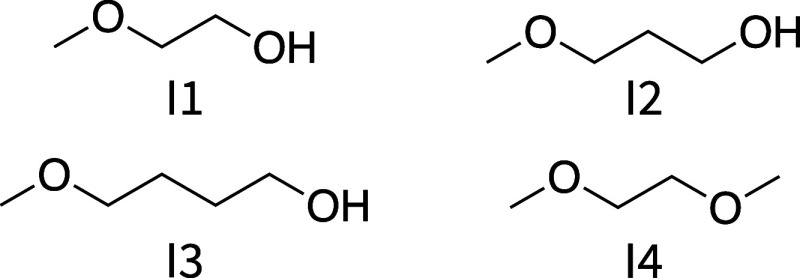
Compound set I.

MD simulations were performed for the molecules in set I (Figure 7): compounds I1, I2, and I3 that feature an intramolecular hydrogen bond forming pseudo 5-membered, 6-membered, or 7-membered rings, respectively, and compound I4 that contains two hydrogen-bond acceptors but no donors. The two main conformers for each compound are depicted in Figure 3A.

The explicit-solvent reference simulations were carried out for 500 ns in three repeats using the same procedure as described above. The simulations with the GNNIS model were performed for 50 ns in ten repeats. To perform these simulations, the OpenMM (version 8.0.0) simulation program^55^ was configured to carry out a vacuum simulation, and the GNN was integrated using the OpenMM-Torch package (version 1.4, https://github.com/openmm/openmm-torch). The OpenMM-Custom Forces classes were used to reimplement the nonbonded interactions to allow for multiple systems to be simulated in parallel. The reference GB-Neck2 simulations were carried out using default OpenMM settings for 1000 ns. For both implicit-solvent simulation methods, the same settings as for the explicit-solvent simulations were employed with the exception that no barostat and no nonbonded cutoff were used.

For compounds I1, I2, and I3, free-energy profiles were computed with direct counting based on the O-H distance histograms. Free energies were further corrected with a Jacobian correction factor of 4 π*r*^2^.^61^ For compound I4, the central torsional dihedral angle was analyzed to construct the histograms and subsequently the free-energy profile. To estimate free-energy differences, the distance or dihedral at the global and second lowest-lying minimum (see Supporting Information Figures S6–S9 for the exact location) of the explicit-solvent simulation was identified, and the Δ*G* between the two minima at these values was calculated for all approaches. Cases where the three repeats of the explicit-solvent reference simulation showed standard deviations >1 *k*_B_*T* were removed from the comparison. This was only the case for compound I3 in HMPA.

### Generation and Minimization of Conformational Ensembles

Minimizations were carried out using the same setup as for the MD simulations. To optimize structures, the L-BFGS algorithm^62^ was used. RDKit^54^ was employed to generate the initial KDG^42^ conformers and MDTraj (version 1.9.7)^63^ was used to prune the minimized conformations based on their heavy-atom RMSD. The selection of a suitable RMSD threshold is not straightforward, as the RMSD metric is sensitive to the size of a molecule.^64,65^ We have, therefore, chosen different thresholds for different molecules, as specified below. Free energies were calculated using normal-mode analysis according to the quasi-RRHO algorithm proposed by Grimme.^44^ Conformers that led to imaginary frequencies were minimized further for up to 100 repeats. Note that some of the minimization did not converge or led to imaginary frequencies in the free-energy calculation. In both cases, the energy was set to infinity, and the conformers were ignored for the follow-up comparisons. The minimization in vacuum and using GB-Neck2 were carried out the same way starting from the same set of random conformers.

#### Compound Sets C and P

**Figure 8:**
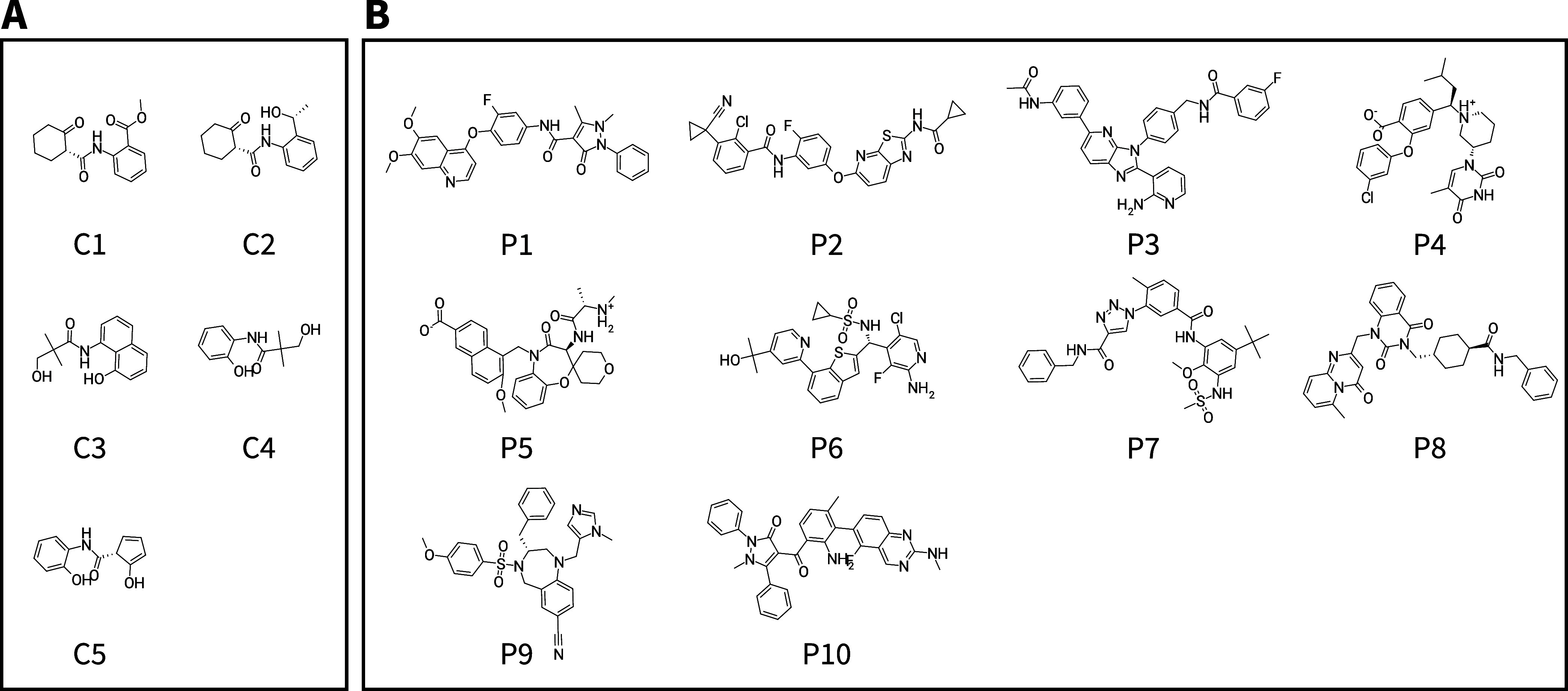
(A) Compound set C. (B) Compound set P.

Two different sets of compounds were chosen for the comparison with explicit-solvent reference simulations (Figure 8): (i) compound set C with five small molecules with a molecular weight <500 Da that can be well sampled and whose conformers can be characterized based on two key dihedral angles, and (ii) compound set P with ten neutral compounds extracted from the Platinum data set^66^ with molecular weights between 500 and 700 Da that feature 5–10 rotatable bonds.

Due to the different size of the compounds, the procedure to generate the conformational ensembles varies slightly between the two sets. For compound set C, 5,120 initial conformers were generated and an RMSD threshold of 0.075 nm was used for pruning. For compound set P, 51,200 initial conformers were generated and an RMSD threshold of 0.15 nm was applied.

During the total ∼2,000,000 minimizations for set P, we realized that the approach yielded unreasonably small energies for two conformers of compound P9 (e.g., the free energy is more than 100 kJ mol^–1^ smaller than the median of the ensemble). In order to exclude conformations that had such high deviations in a systematic way, all conformations with free energies more than four standard deviations away from the ensemble median were excluded. Further, for compound P10, which is an atropisomer, only conformers of the correct isomer were considered for the comparison to the explicit-solvent reference simulation.

The reference ensembles were extracted from MD simulations performed using the enhanced sampling method REST2,^67^ following the setup described by Waibl et al.^68^ GROMACS^69−71^ 2023 with the PLUMED^72−74^ 2.9.1 plugin was used. Simulations were performed for 100 and 200 ns for set C and P, respectively. Eight replicas and a quadratic scheme to distribute the scaling factors between 1 and 0.125 were applied.

The comparison to explicit-solvent reference simulations was conducted by taking the final conformers from the minimization workflow and considering them as separate conformational states. The reference simulation was then used to assign a population to each state by assigning each frame of the reference simulation to the closest conformer below an RMSD threshold of 0.1 nm. These populations were converted to free energies and compared to the free energy predicted by the GNNIS approach. The same procedure was performed with the conformer ensembles minimized in a vacuum and using GB-Neck2.

For quantification, a confusion matrix (see Supporting Information Figure S19) was defined by separating the correlation plots into four bins using a cutoff at 1 *k*_B_*T*:

- True positive: *G*_GNNIS_ < 1 *k*_B_*T* and *G*_explicit_ < 1 *k*_B_*T*
- True negative: *G*_GNNIS_ > 1 *k*_B_*T* and *G*_explicit_ > 1 *k*_B_*T*
- False positive: *G*_GNNIS_ < 1 *k*_B_*T* and *G*_explicit_ > 1 *k*_B_*T*
- False negative: *G*_GNNIS_ > 1 *k*_B_*T* and *G*_explicit_ < 1 *k*_B_*T*.

From this, balanced accuracy was calculated using the SciPy library.^75^ Note that for some minimizations in vacuum and using GB-Neck2, no true negatives were found. In these cases, the true negative rate was set to zero for the calculation of the balanced accuracy.

#### NMR Measurements

Experimental ^1^H NMR measurements for compound I1 were conducted in deuterated solvents at room temperature. The measured NMR spectra, as well as the experimental details, are given in Supporting Information Section S3.

The NMR data for compound I4 recorded at 40 °C were taken from ref (45). The experimental observable *J*_tot_ was computed by measuring the distance between the two outer peaks of the multiplet (see Figure 5B) using the MestReNova software (version 14.3.3). The spectra and MestReNova files are available in the ETH Research Collection (DOI: 10.3929/ethz-b-000710355).

The experimental data for the 22 molecular balances were taken from ref (46).

#### Conformational Ensembles of Compounds I1, I4, and Molecular Balances

For the minimization, 256 initial KDG conformers were generated and an RMSD threshold of 0.05 nm was used. The dihedral angle around the central bond shown in Figure 5A was analyzed. Dihedral angles between −2.1 and 2.1 rad were categorized as the gauche-conformer, while all other dihedral angles were classified as the trans-conformer. Next, the total populations of these two states were evaluated by weighting each state according to its predicted free energy.

For the 22 molecular balances, 5,120 KDG conformers were generated and minimized. The conformers were divided into two sets based on the rotation state of the ketone group (see Figure 6A) and RMSD pruned using a threshold of 0.075 nm. The populations of the two groups were assessed based on the free energies of the conformers, and the difference in free energy between the two rotamers was calculated. To compare with experimental data, the mean of the predicted values for each conformer was subtracted from the predictions, while the mean of the experimental values was subtracted from the experimental data.

## Data Availability

The open-source code is available on GitHub (https://github.com/rinikerlab/GNNImplicitSolvent). All data required for the training and testing of the GNN is made freely available in the ETH Research Collection (DOI: 10.3929/ethz-b-000710355). All other data points (e.g., trajectories, minimized conformers, etc.) are available upon reasonable request.

## Supporting Information Available

Description of the GNN architecture, physical properties of solvents, details of NMR measurements, GB model comparison, simulation timings, technical description of simulation setup, timings of the minimization approach, and additional figures (PDF)
