# Supplementary material for: Rapid Access to Small Molecule Conformational Ensembles in Organic Solvents Enabled by Graph Neural Network-Based Implicit Solvent Model
Source: J Am Chem Soc. 2025 Apr 10;147(16):13264–75. doi: 10.1021/jacs.4c17622 (PMC12022995; doi:10.1021/jacs.4c17622)
Supplement: Supplementary file 1 — ja4c17622_si_001.pdf [file ja4c17622_si_001.pdf]

SUPPORTING INFORMATION
Rapid Access to Small Molecule Conformational Ensembles in Organic Solvents Enabled by Graph Neural Network Based Implicit Solvent Model

Paul Katzberger,[a] Lea Marie Hauswirth,[a] Antonia S. Kuhn,[a] Gregory A. Landrum,[a] and Sereina Riniker[a]*

[a] *Department of Chemistry and Applied Biosciences, ETH Zürich, Vladimir-Prelog-Weg 2, 8093 Zürich, Switzerland. E-mail: sriniker@ethz.ch*

Contents

S1 Graph Neural Network **S2**

S2 Properties of the 39 Solvents **S3**

S3 NMR Measurements **S4**

S4 GB Model Comparison **S4**

S5 Timings for the Simulations **S5**

S6 Simulation of multiple systems in parallel **S6**

S7 Timings for Minimization Approach **S7**

S8 Additional Figures **S8**

S8.1 Training Validation S8
S8.2 Prospective MD Simulations S9
S8.3 Rapid Assessment of Conformational Ensembles S18
S8.4 Comparison with Experimental Observables S26
S8.5 Molecular Balances S27

S1 Graph Neural Network

A schematic representation of the graph neural network (GNN) is shown in Figure 1A in the main text. The architecture can be described as follows:

First, the GB-Neck2 algorithm[1] is used to calculate the GB radii R_i for each atom i. The GB radii, partial charges, and the GB-Neck2 van der Waals radii of the atoms are then concatenated to form the input features for each node. For the message passing step, the node features of two connected atoms ($r_{ij} < 0.6\,nm$) and the distances r_{ij} encoded by a Bessel function (with $n = 20$) are concatenated and processed by the message layer which consist of the following sequence: [Linear(64), SiLU, Linear(64), SiLU]. For the following node-wise operations, the messages are aggregated by summation and concatenated with the solvent embedding consisting of a learnable feature vector of length 64 for each solvent. During the first two passes, the layer configuration is [Linear(128), SiLU, Dropout, Linear(64)] while for the final readout [Linear(128), SiLU, Dropout, Linear(2)] is applied.

The output x, which is a vector of size 2 for each atom, is split into two components: x_0 and x_1. The first component, x_0, is used to scale the GB radii. These scaled radii are then applied in the generalized Born formula to calculate the electrostatic component of the energy:

$$R_i^s = R_i * (0.1 + S(x_{0i}) * 1.8)$$
$$\Delta G^{elec} = -\frac{1}{2}\left(\frac{1}{\varepsilon_{in}} - \frac{1}{\varepsilon_{out}}\right)\sum_{i,j}\frac{q_i q_j}{\sqrt{r_{ij}^2 + R_i^s R_j^s \exp\left(\frac{-r_{ij}^2}{4R_i^s R_j^s}\right)}},$$

where S represents the sigmoid function and ε_{out} the dielectric permittivity of the specific solvent.

The second component, x_1, is used to scale a solvent-specific learnable surface tension coefficient γ_s to predict the nonpolar contribution:

$$\Delta G^{np} = \gamma_s * S(x_{1i}) * (r_{VdW_i} + 0.0195141 + 0.14)^2$$

The final energy is calculated as the sum of both free energies. The model is then trained on the derivative of the total free energies with respect to the atom positions (i.e., the predicted force for each atom).

S2 Properties of the 39 Solvents

The density and dielectric permittivity (ε) data for all 39 solvents was collected from the Handbook of Chemistry and Physics[2] if not indicated otherwise.

Solvent	Density [$kg\,L^{-1}$]	ε
Water	1.0	78.5
Chloroform	1.49	4.81
Methanol	0.792	33.0
DMSO	1.1	47.24
DMPU[3]	1.06	36.12
Diethyl Ether	0.7134	4.27
Ethanol	0.78945	25.3
DMF	0.948	38.25
DCM	1.3266	8.93
Toluene	0.8623	2.38
Benzene	0.8765	2.28
Hexane	0.6606	1.88
Acetonitrile	0.786	36.64
Acetone	0.7845	21.01
Acetic Acid	1.049	6.2
1,4-Dioxane	1.033	2.22
Nitrobenzene	1.199	35.6
HMPA[4]	1.03	29.6
MTBE[5]	0.7404	4.5
IPA	0.786	20.18
Hexafluorobenzene	2.03	2.03
Pyridine	0.9819	13.26
THF	0.8876	7.52
Ethyl Acetate	0.902	6.2
Sulfolane[6]	1.261	43.3
Nitromethane	1.1371	37.27
Butyl Formate	0.872	6.1
NMP	1.028	32.55
Octanol	0.83	10.3
Cyclohexane	0.7739	2.024
Glycerin	1.261	46.53
Carbon Tetrachloride	1.5867	2.24
DME	0.8683	7.3
2-Nitropropane	0.9821	26.74
Trifluorotoluene[7]	1.19	9.22
Hexafluoroacetone	1.32	2.104
Propionitrile	0.772	29.7
Benzonitrile	1.0	25.9
Oxylol	0.88	2.56

S3 NMR Measurements

For each solvent, a ^{1}H (16 scans, sw 16.4 ppm, td 80126, o1p 6.0 ppm, aq 4.9 s, d1 0.01 s) NMR spectrum of compound I1 was acquired for each solvent on a Bruker AVANCE III 500 MHz spectrometer equipped with a Bruker Broad Band probe with xyz-gradients at 25.0 °C.

NMR data is freely available in the ETH Research Collection (DOI: 10.3929/ethz-b-000710355).

S4 GB Model Comparison

The difference between two different GB-based models GB-Neck2[1] and GB-OBC[8] was analyzed using set I. The same analysis as for the explicit-solvent reference was performed for the GB-OBC model. The correlation of the predicted free-energy differences between the GB-OBC and explicit-solvent reference are shown in Figure S1.

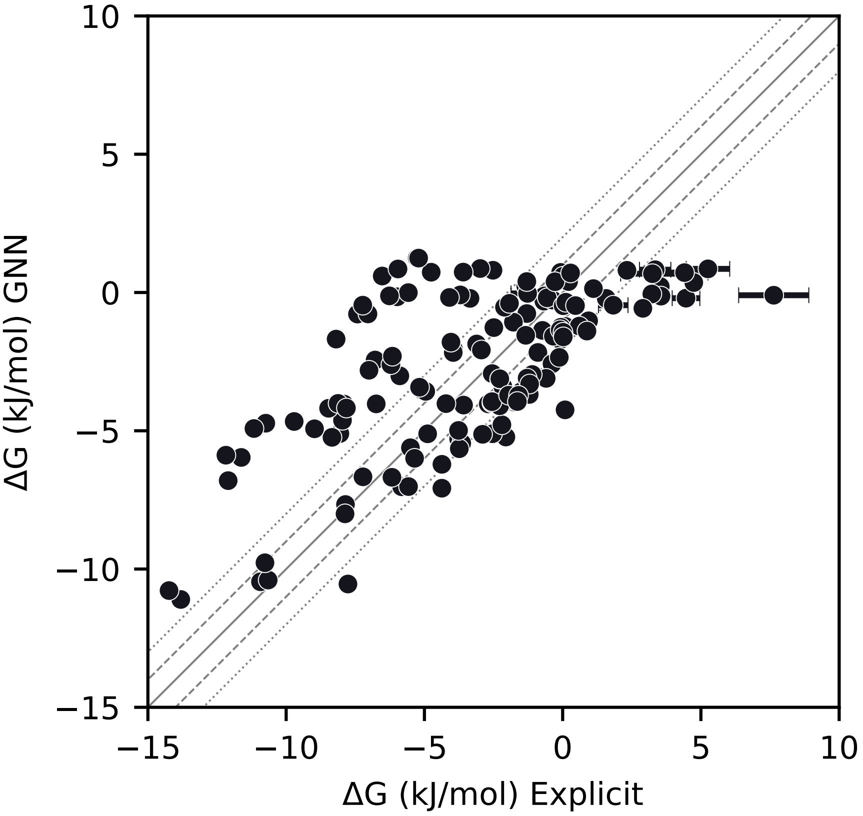

Figure S1: Comparison of the GB-OBC implicit solvent model (1 x 1000 ns) with the explicit-solvent reference (3 x 500 ns). The comparison of the free-energy difference ΔG between the lowest lying minima of the closed and opened intramolecular hydrogen bond for the GB-OBC and explicit-solvent simulation is shown. Error bars indicate the standard deviation across the simulation replicates. Deviations below 0.5 kJ mol^{-1} are omitted for clarity.

Overall, the results are qualitatively similar to the GB-Neck2 results, with significant deviations be-

tween the implicit solvent model and the explicit-solvent reference. Comparing the GB-OBC results with the results of GB-Neck2 further highlights that the two GB-based methods only differ slightly (Figure S2).

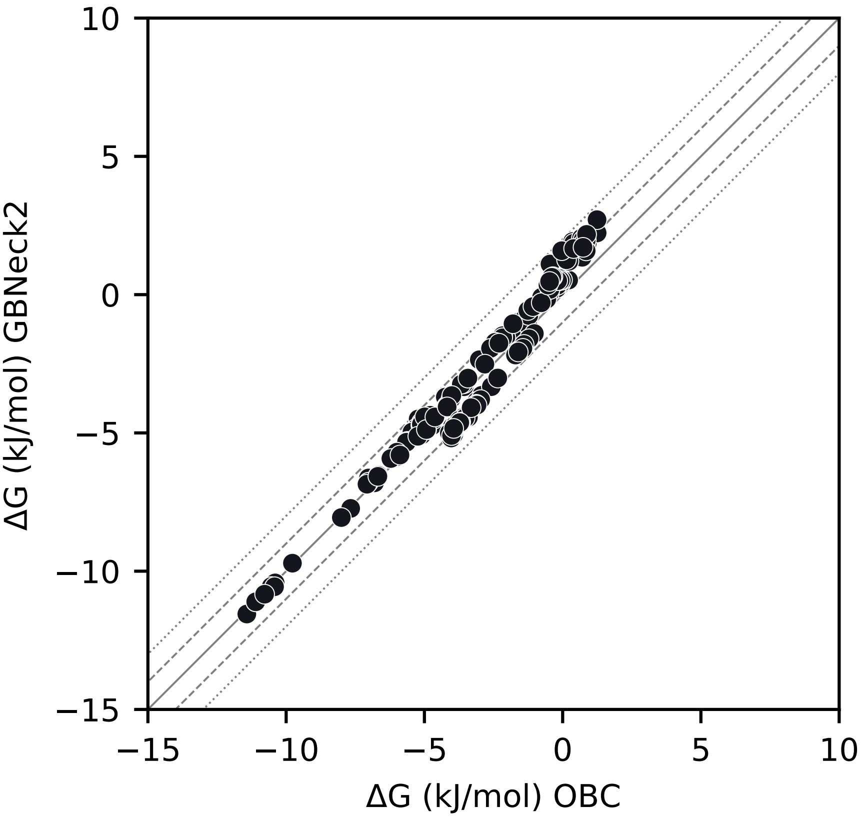

Figure S2: Comparison of the GB-OBC implicit solvent model (1 x 1000 ns) with the GB-Neck2 implicit solvent model (1 x 1000 ns). The comparison of the free-energy difference ΔG between the lowest lying minima of the closed and opened intramolecular hydrogen bond for the GB-OBC and GB-Neck2 simulation is shown. Error bars indicate the standard deviation across the simulation replicates. Deviations below 0.5 kJ mol^{-1} are omitted for clarity.

S5 Timings for the Simulations

To asses how the speed of the simulation is related to the size of the studied molecular system. We took the first three molecules studied in set I, which take the general form COC_NO and continued to grow the alkyl chain up to N = 50. First, the systems were simulated with only one replicate. This revealed that all compounds had approximately the same simulation speed regardless of the system size (see Figure S3). The reason for this is that the simulation of only one system does not fully utilize the GPU.

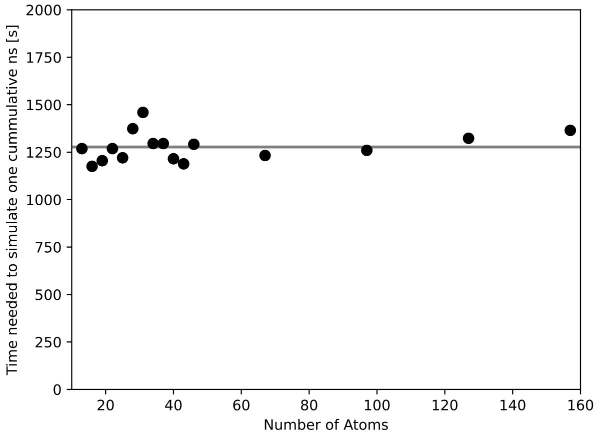

Figure S3: Simulation speed of GNNIS simulations of one system.

In contrast, when multiple systems were studied in parallel on the same GPU (i.e., 500 replicates for systems of $N \leq 20$ and 100 replicates for $N \geq 30$) a clear relationship between speed and system size becomes apparent (see Figure S4). Within the studied set of systems the observed scaling seems to agree well with a *NlogN* scaling which would also be expected for explicit solvent simulations (see grey line in Figure S4).

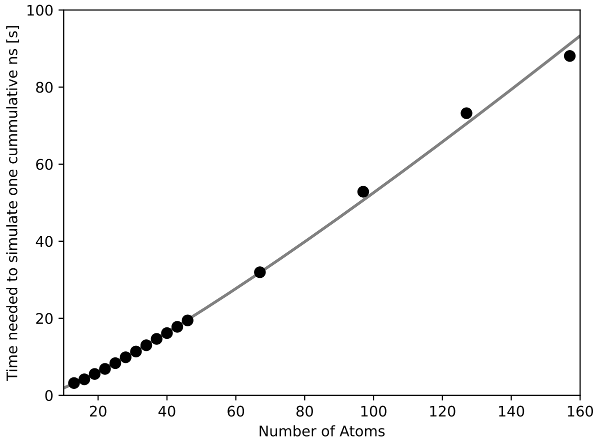

Figure S4: Simulation speed of GNNIS simulations of multiple replicates of one system. The grey line indicates a fitted function of $a \cdot N_A \log(N_A) + b$, where N_A is the number of atoms in the system.

S6 Simulation of multiple systems in parallel

The simulation of multiple systems simultaneously was already employed in our previous work[9]. Briefly, rather than simulating one system, multiple systems of non-interacting molecules are run in parallel. On a technical level this is made possible by the `CustomNonbondedForce` class provided by the OpenMM software package. First the standard non-bonded forces are reimplemented using this class and then the pre-implemented `addInteractionGroup` function is used to only add this force contribution to the atoms of one replicate. In essence, this procedure allows the OpenMM software to be used to run a simulation

of multiple molecules that do not interact with each other and can hence be treated as independent simulations. If someone is interested in the exact implementation, we recommend looking at the code made available on GitHub (`https://github.com/rinikerlab/GNNImplicitSolvent`).

S7 Timings for Minimization Approach

The reference explicit-solvent MD simulations required roughly 14 hours with 8 CPU cores and a NVIDIA RTX 4090 consumer GPU. The timings of the proposed workflow for compound C1 for the same computational resources are shown in Table S2.

Table S2: Time necessary for the minimization approach for compound C1 in water split up into its components performed with 8 CPU cores and a NVIDIA RTX 4090 GPU.

KDG ensemble generation:	74 s
OpenFF 2.0.0 + GNNIS model minimization:	202 s
Entropy calculation:	215 s

Notably, only the OpenFF 2.0.0 + GNNIS model minimization can fully use the GPU. The KDG algorithm is only performed on the CPU. Similarly, the entropy calculation based on Grimme's quasi-RRHO approach[10] requires the calculation of the Hessian. While the analytical solution would be fast to obtain, OpenMM (the software used in this project) has no implementation for this, requiring the numerical calculation of the Hessian, which is computationally inefficient, as a huge overhead is created by constant CPU – GPU communication. In principle, this step could also be performed on the CPU, with little change in performance.

Timings for the key OpenFF 2.0.0 + GNNIS model minimization step of the conformers of compound sets C and P are shown in Table S3. As a point of reference, a vacuum minimization has been added.

Table S3: Benchmark of OpenFF 2.0.0 + GNNIS model minimization times for the two compound sets C (8 CPU cores + NVIDIA RTX 3090 GPU) and P (8 CPU cores + NVIDIA RTX 4090 GPU).

Compound Set	Vacuum [s per conformer]	GNNIS [s per conformer]
C	0.01	0.05
P	0.03	0.11

S8 Additional Figures

S8.1 Training Validation

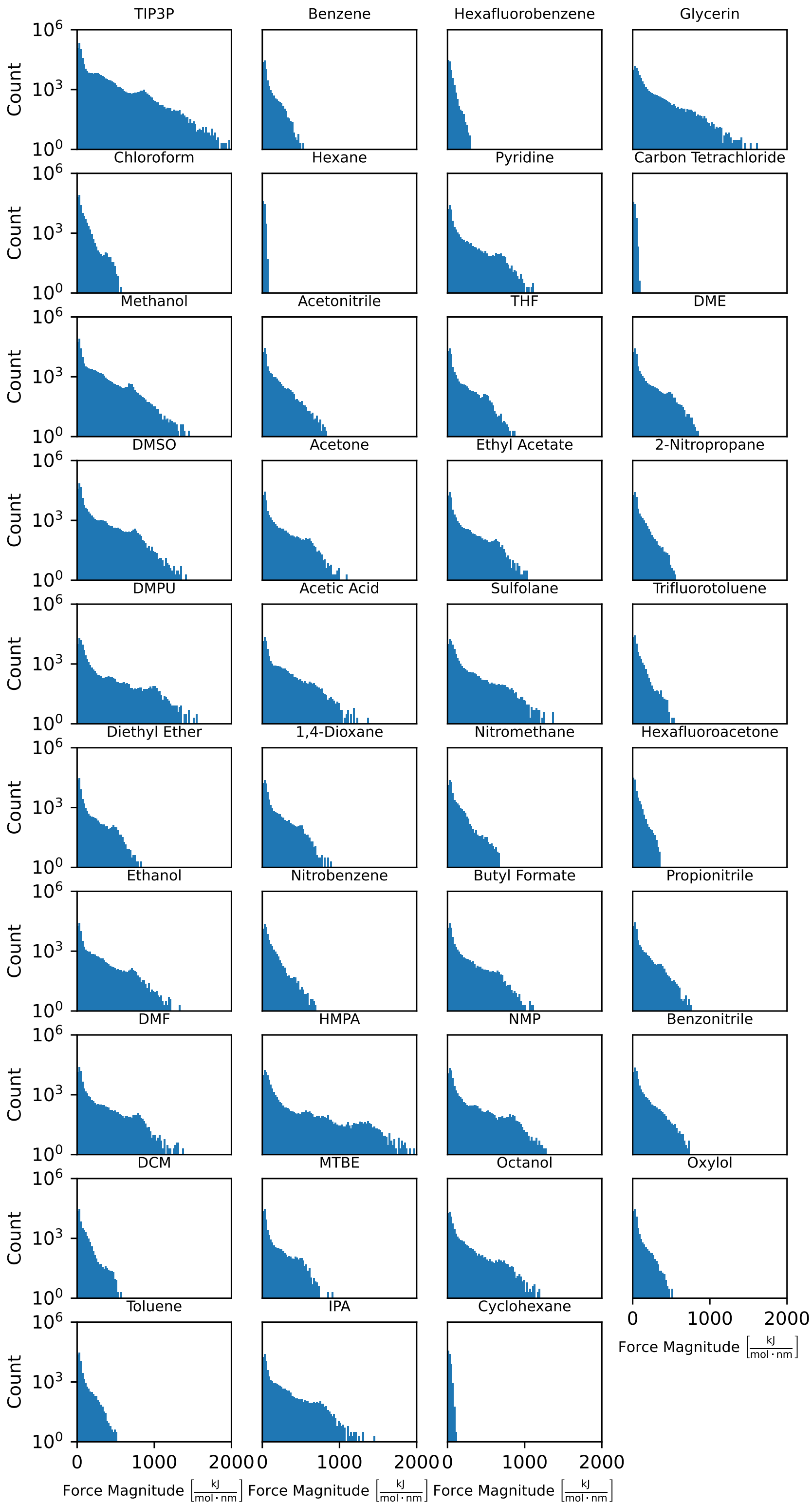

Figure S5: Distributions of the force magnitude from the explicit-solvent reference for all solvents.

S8.2 Prospective MD Simulations

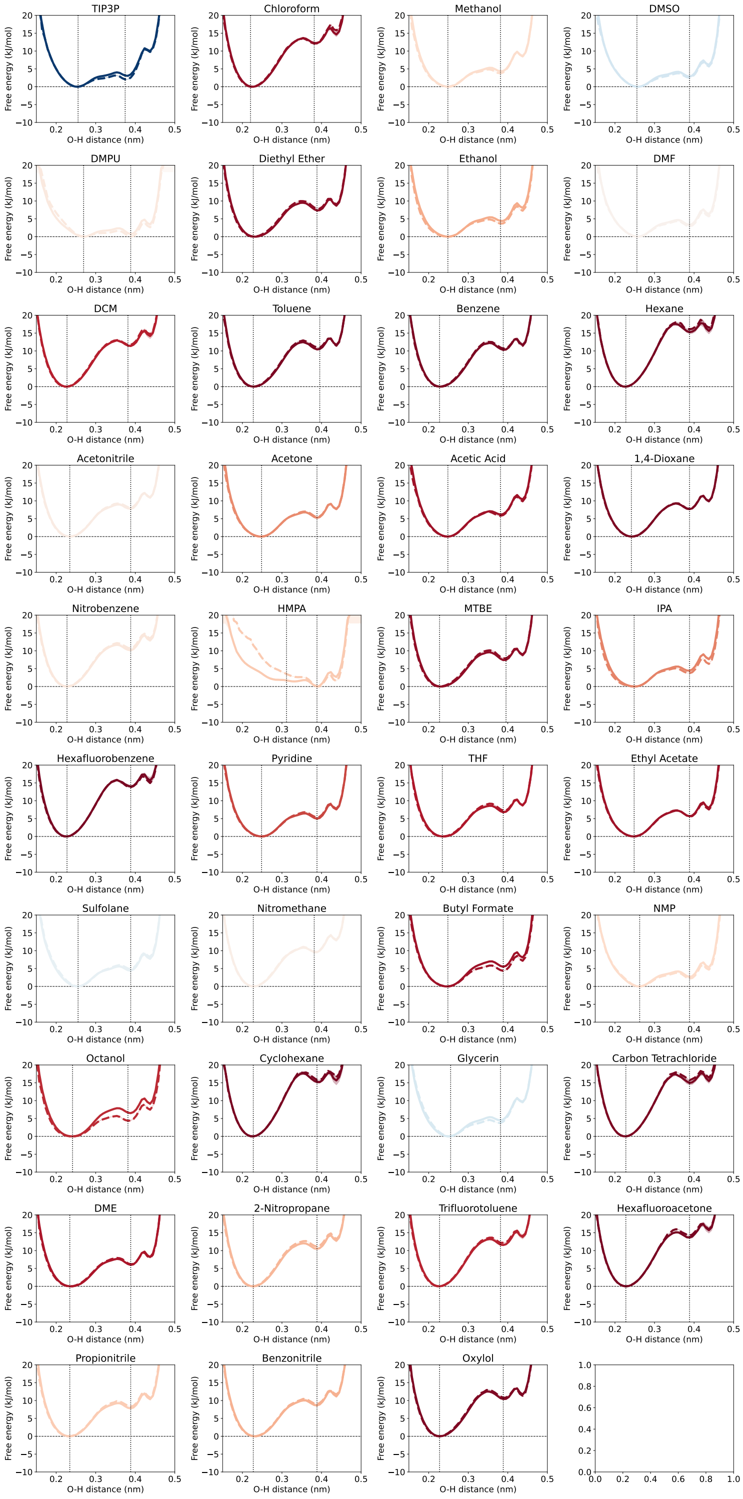

Figure S6: Comparison of the GNNIS model (solid, 10 x 50 ns) with the explicit-solvent reference (dashed, 3 x 500 ns). The free-energy profile of the opening of the intramolecular hydrogen bond of compound I1 simulated in all solvents is shown. The global minimum and the lowest-lying local minimum for the explicit-solvent simulation is indicated by the black dashed line. The shaded area indicates the variability over the GNNIS simulation replicates.

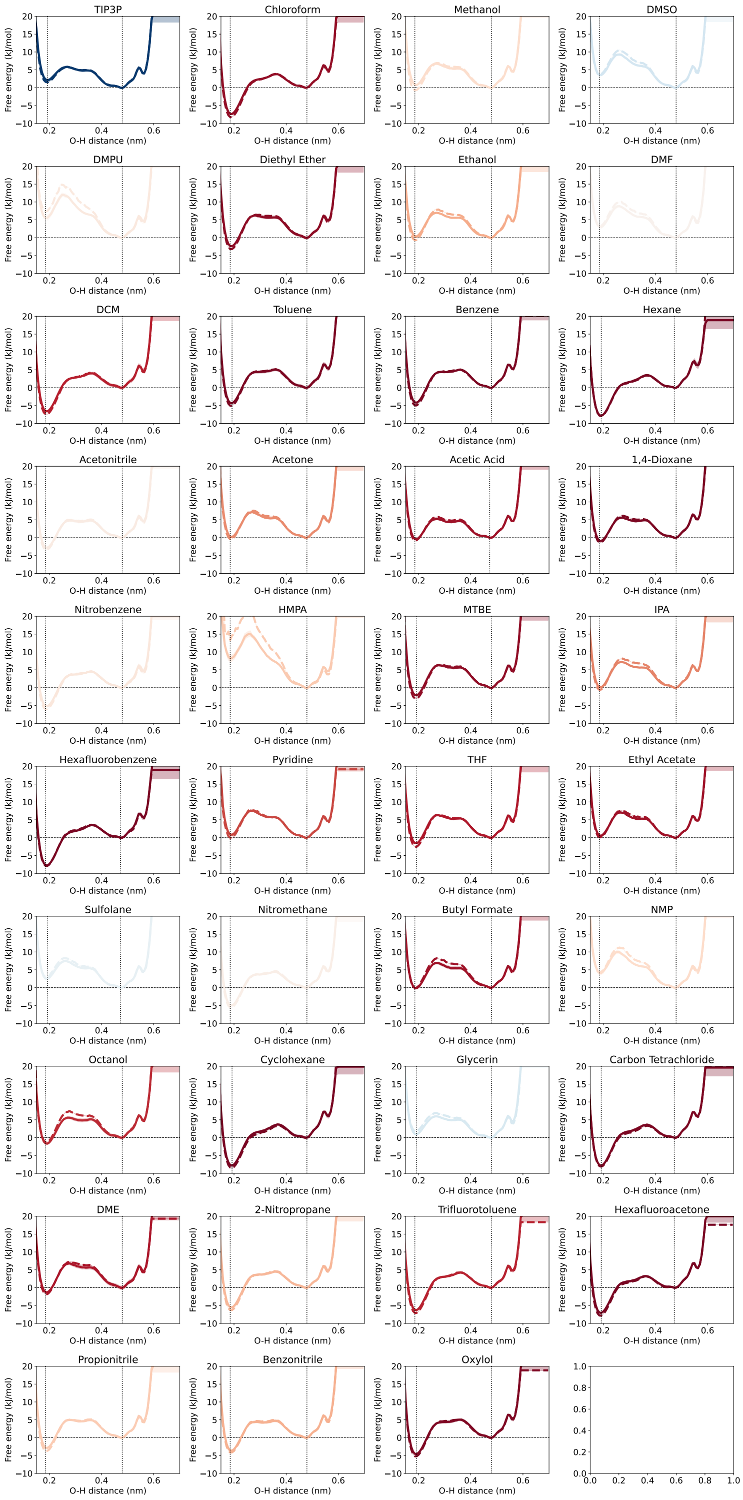

Figure S7: Comparison of the GNNIS model (solid, 10 x 50 ns) with the explicit-solvent reference (dashed, 3 x 500 ns). The free-energy profile of the opening of the intramolecular hydrogen bond of compound I2 simulated in all solvents is shown. The global minimum and the lowest-lying local minimum for the explicit-solvent simulation is indicated by the black dashed line. The shaded area indicates the variability over the GNNIS simulation replicates.

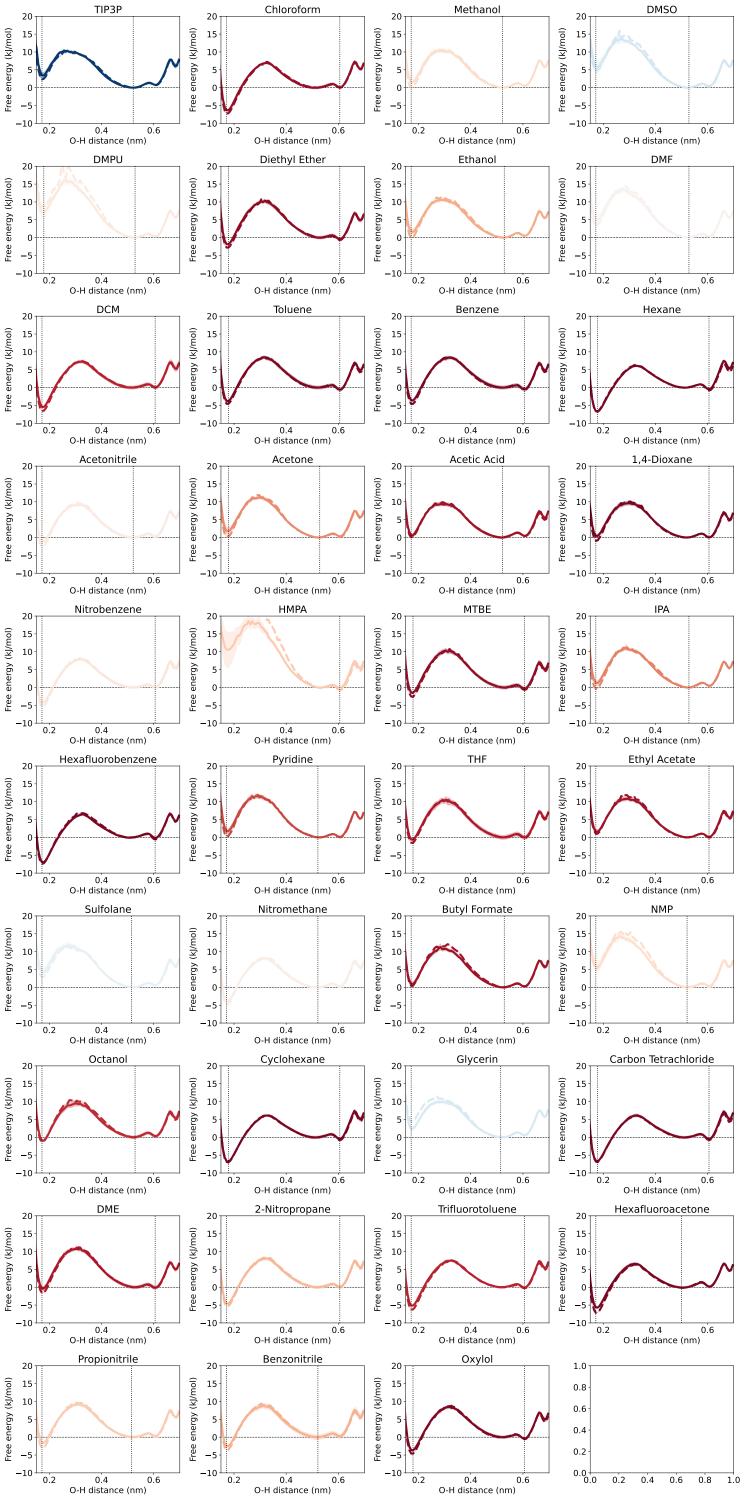

Figure S8: Comparison of the GNNIS model (solid, 10 x 50 ns) with the explicit-solvent reference (dashed, 3 x 500 ns). The free-energy profile of the opening of the intramolecular hydrogen bond of compound I3 simulated in all solvents is shown. The global minimum and the lowest-lying local minimum for the explicit-solvent simulation is indicated by the black dashed line. The shaded area indicates the variability over the GNNIS simulation replicates.

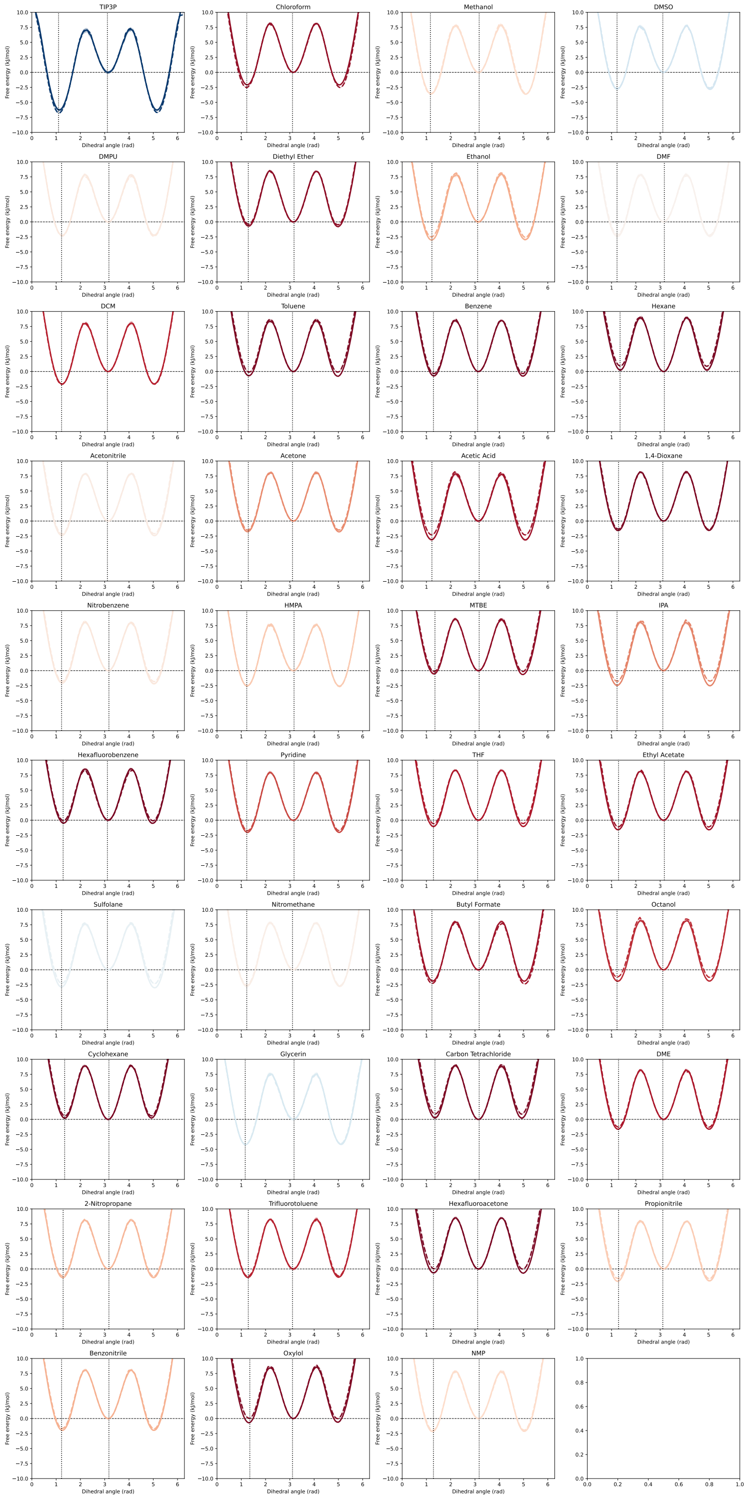

Figure S9: Comparison of the GNNIS model (solid, 10 x 50 ns) with the explicit-solvent reference (dashed, 3 x 500 ns). The free-energy profile of the opening of the intramolecular hydrogen bond of compound I4 simulated in all solvents is shown. The global minimum and the lowest-lying local minimum for the explicit-solvent simulation is indicated by the black dashed line. The shaded area indicates the variability over the GNNIS simulation replicates.

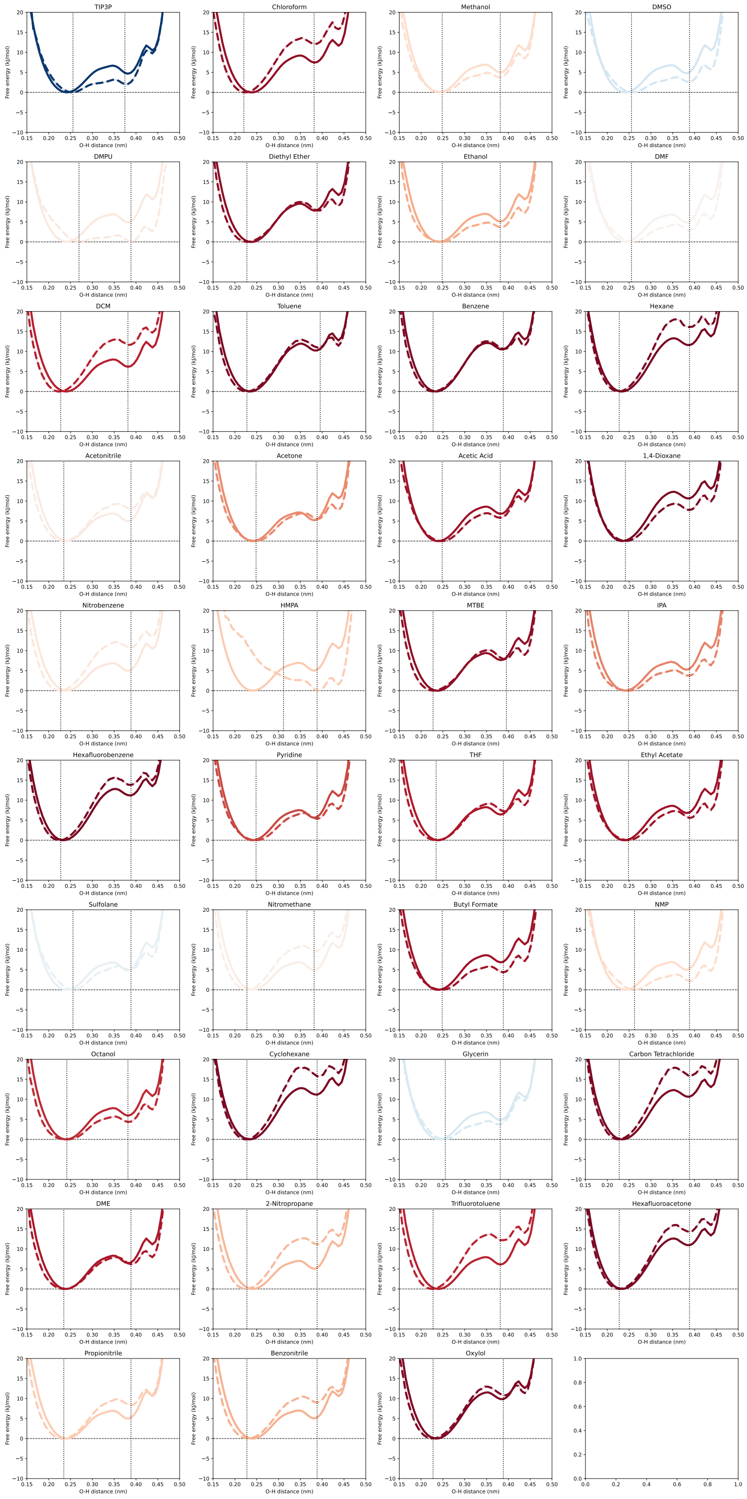

Figure S10: Comparison of the GB-Neck2 implicit solvent model (solid, 1 x 1000 ns) with the explicit-solvent reference (dashed, 3 x 500 ns). The free-energy profile of the opening of the intramolecular hydrogen bond of compound I1 simulated in all solvents is shown. The global minimum and the lowest-lying local minimum for the explicit-solvent simulation is indicated by the black dashed line.

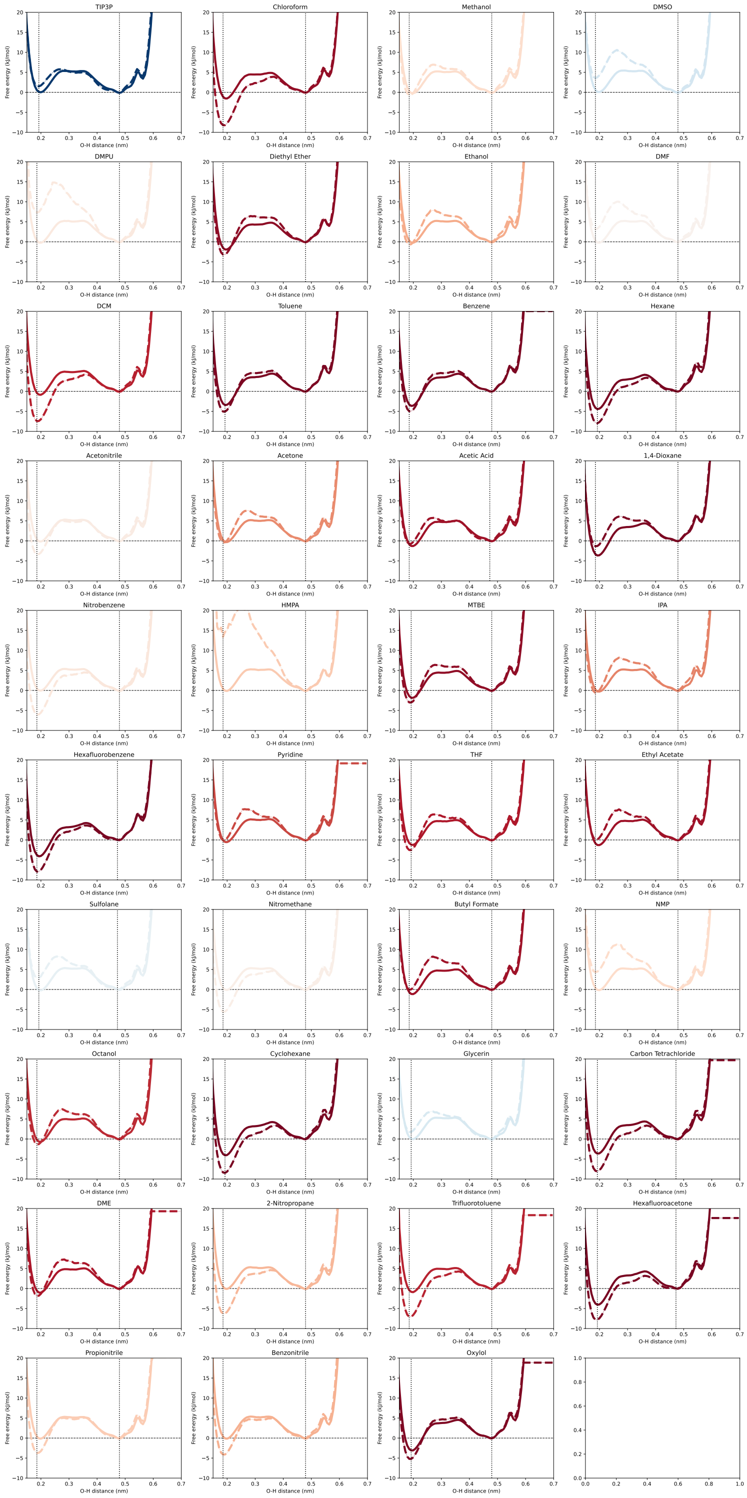

Figure S11: Comparison of the GB-Neck2 implicit solvent model (solid, 1 x 1000 ns) with the explicit-solvent reference (dashed, 3 x 500 ns). The free-energy profile of the opening of the intramolecular hydrogen bond of compound I2 simulated in all solvents is shown. The global minimum and the lowest-lying local minimum for the explicit-solvent simulation is indicated by the black dashed line.

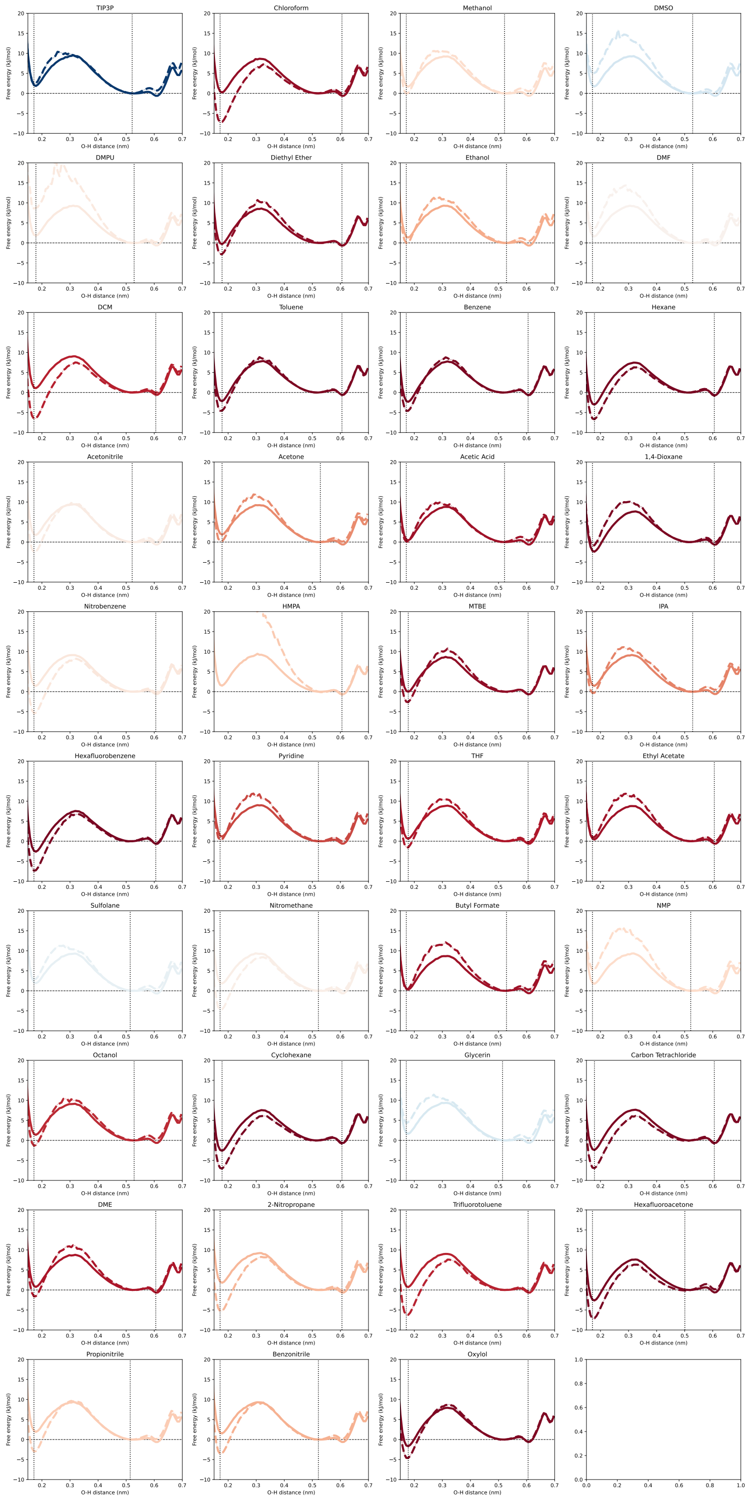

Figure S12: Comparison of the GB-Neck2 implicit solvent model (solid, 1 x 1000 ns) with the explicit-solvent reference (dashed, 3 x 500 ns). The free-energy profile of the opening of the intramolecular hydrogen bond of compound I3 simulated in all solvents is shown. The global minimum and the lowest-lying local minimum for the explicit-solvent simulation is indicated by the black dashed line.

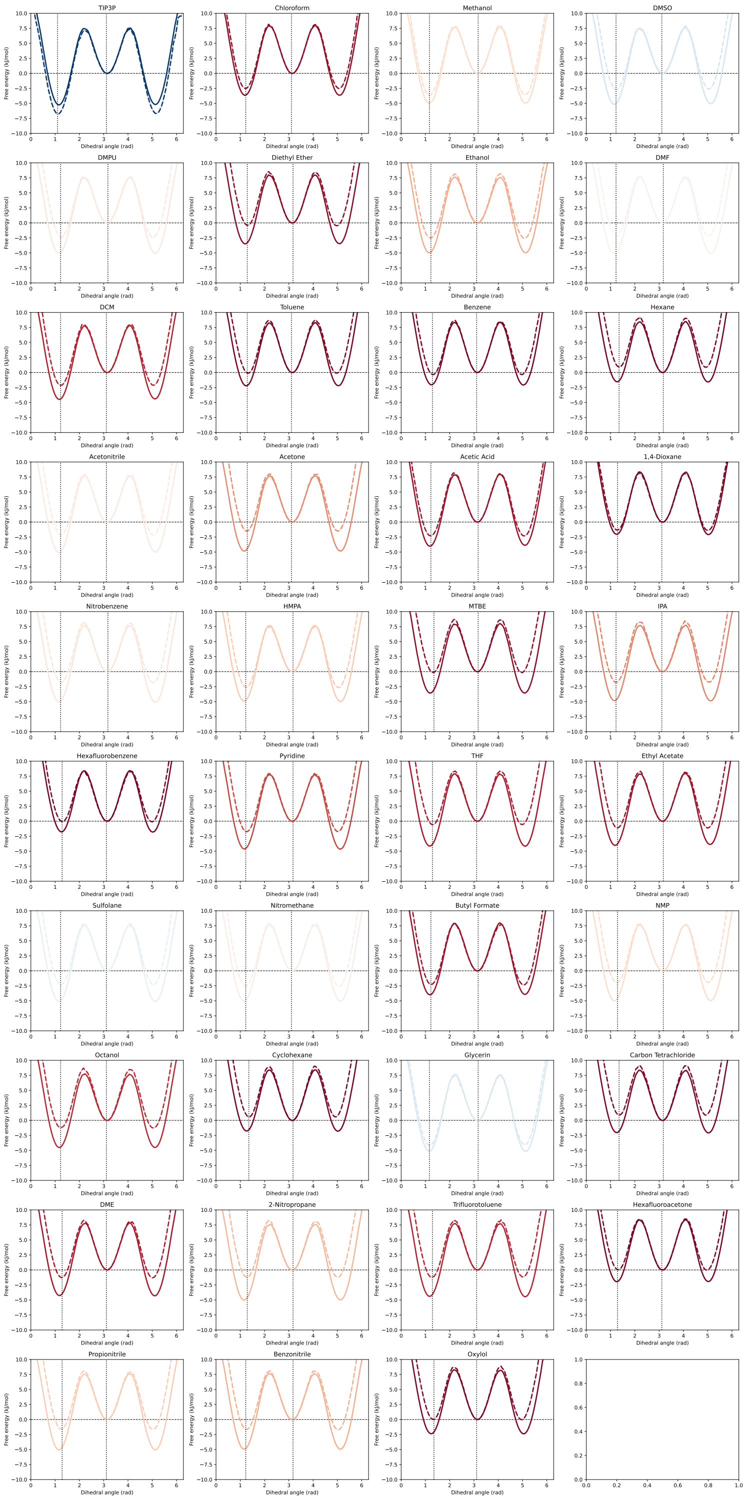

Figure S13: Comparison of the GB-Neck2 implicit solvent model (solid, 1 x 1000 ns) with the explicit-solvent reference (dashed, 3 x 500 ns). The free-energy profile of the opening of the intramolecular hydrogen bond of compound I4 simulated in all solvents is shown. The global minimum and the lowest-lying local minimum for the explicit-solvent simulation is indicated by the black dashed line.

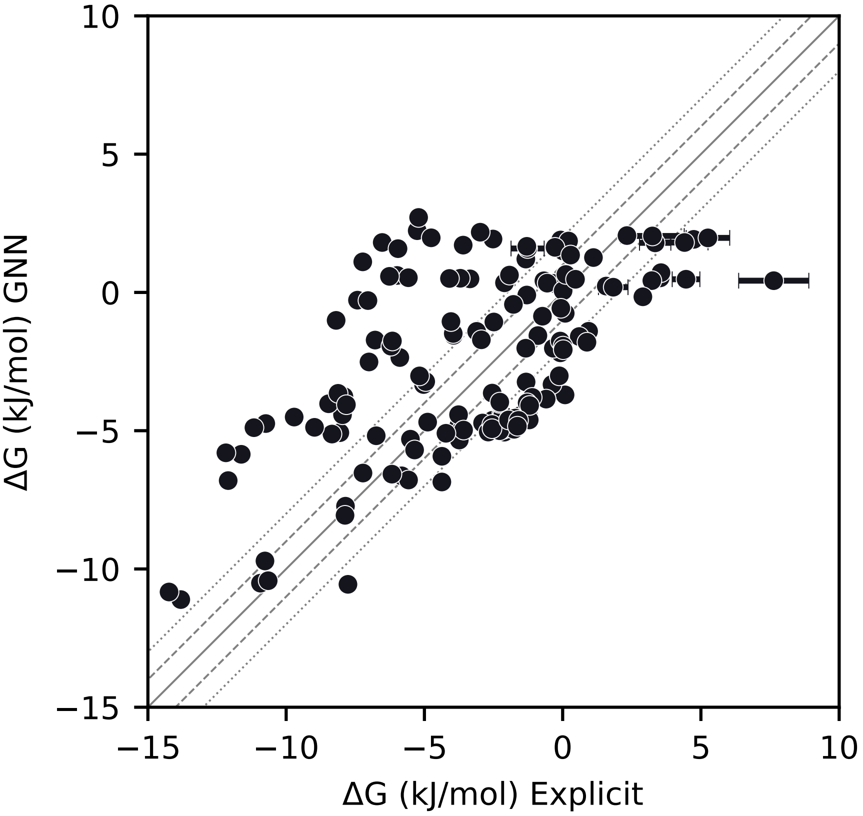

Figure S14: Comparison of the GB-Neck2 implicit solvent model (1 x 1000 ns) with the explicit-solvent reference (3 x 500 ns) The comparison of the free-energy difference ΔG between the global minimum and the lowest-lying local minimum for the GB-Neck2 and explicit-solvent simulation is shown. Error bars indicate the standard deviation across the simulation replicates. Deviations below $0.5\,\mathrm{kJ\,mol^{-1}}$ are omitted for clarity.

S8.3 Rapid Assessment of Conformational Ensembles

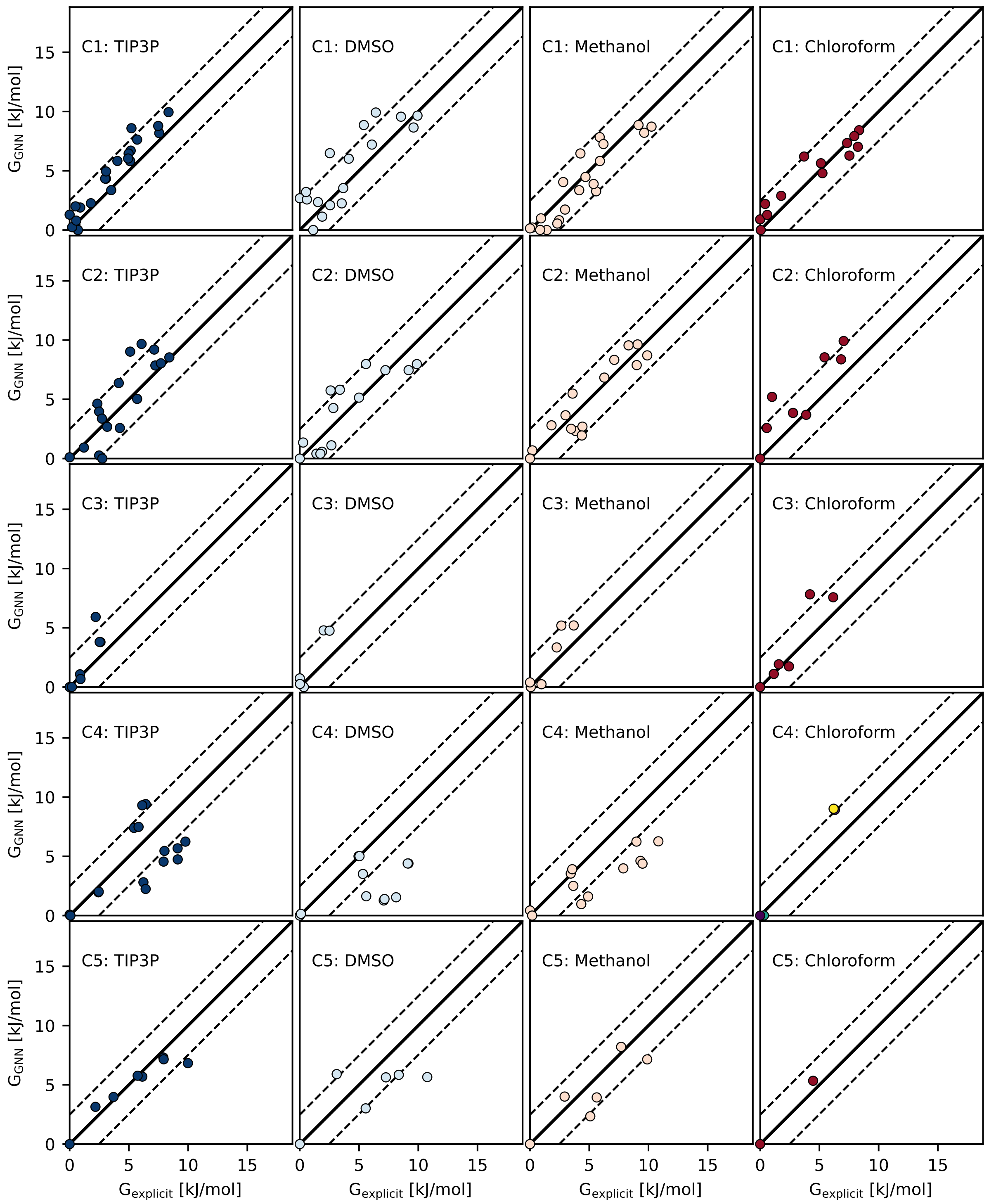

Figure S15: Comparison of the conformational ensemble minimized using the GNNIS model with the explicit-solvent REST2 simulation for compound set C.

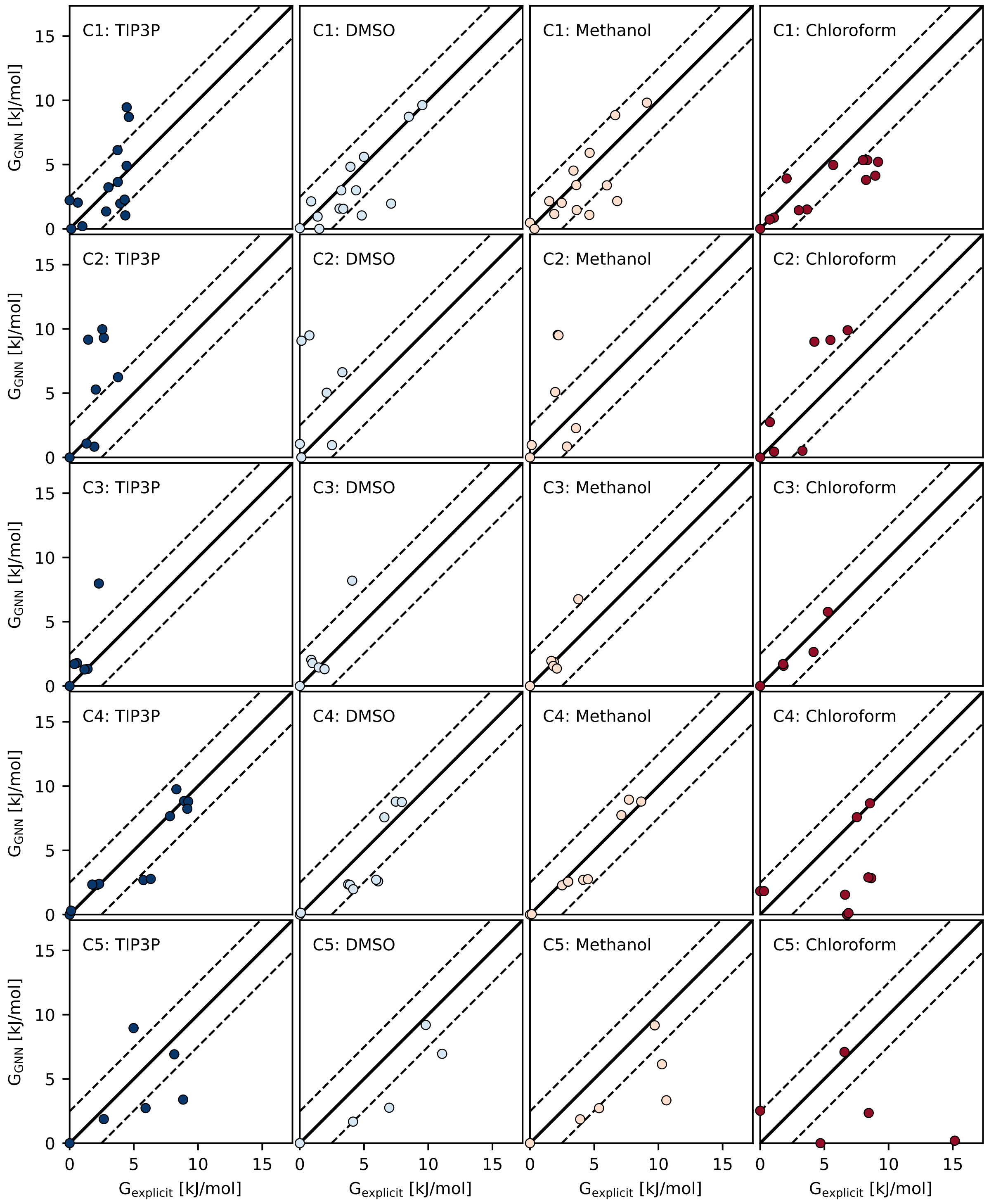

Figure S16: Comparison of the conformational ensemble minimized using the GB-Neck2 implicit solvent model with the explicit-solvent REST2 simulation for compound set C.

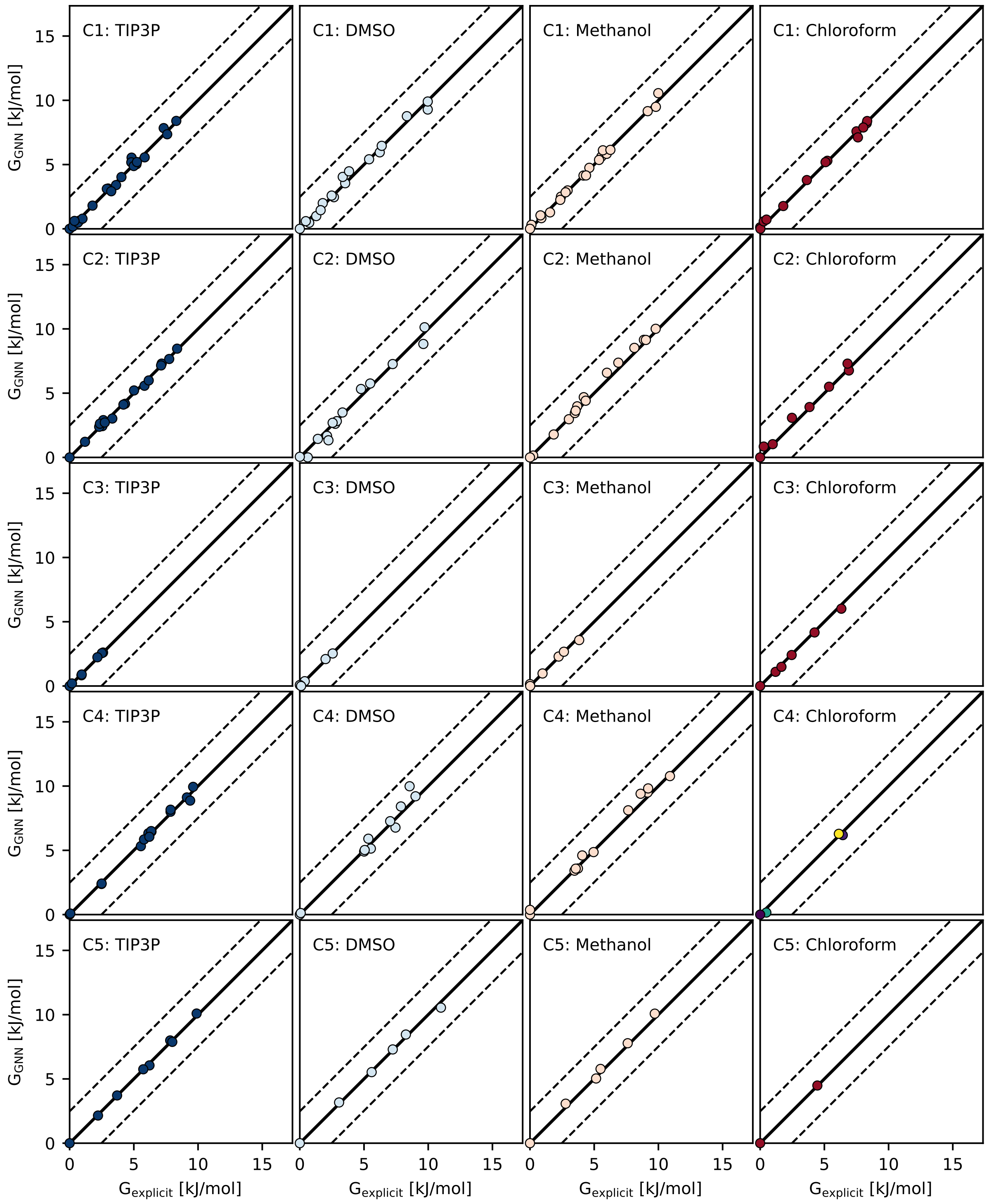

Figure S17: Comparison of the first half of the explicit-solvent REST2 simulations with the second half for compound set C.

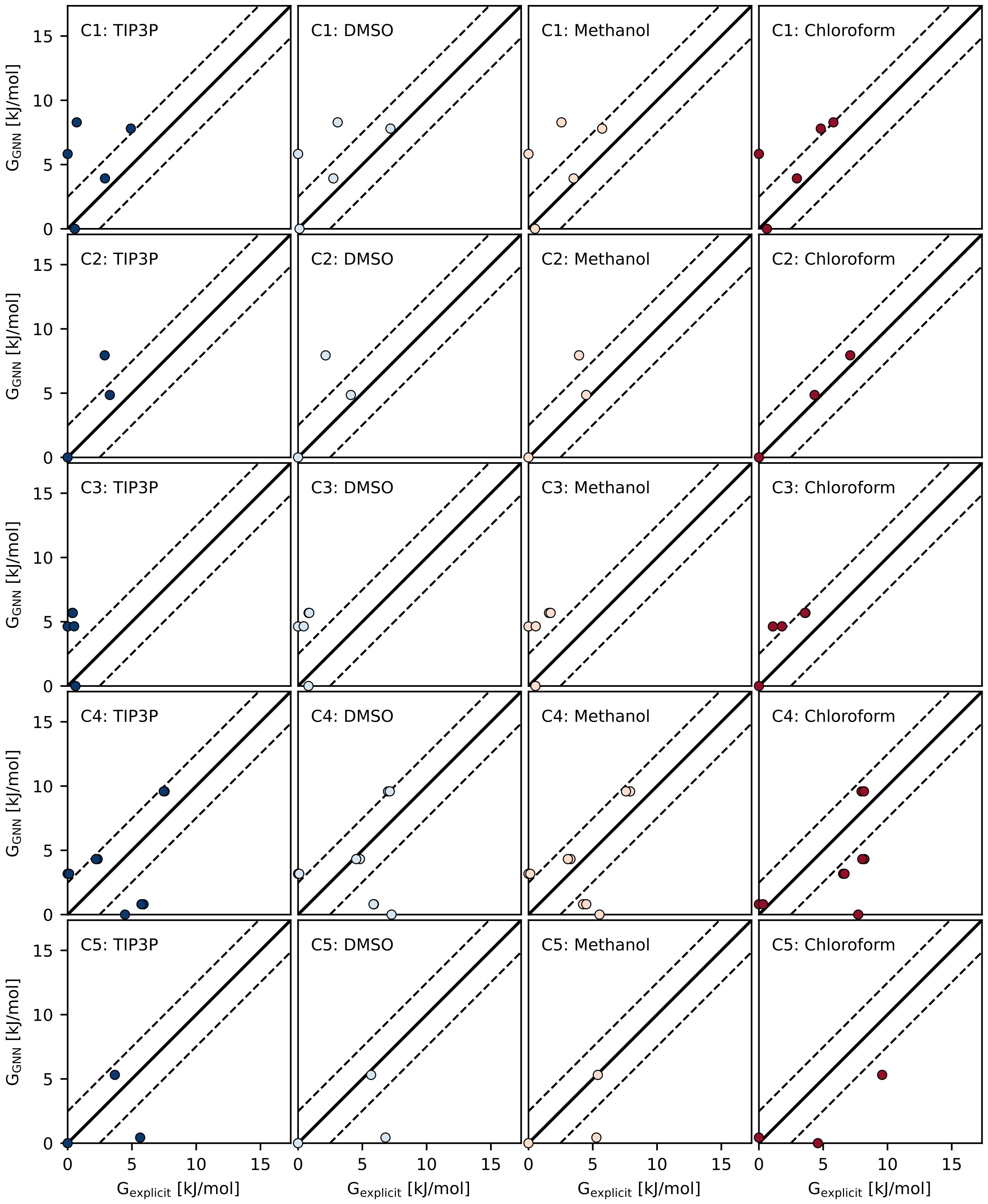

Figure S18: Comparison of the conformational ensemble minimized in vacuum with the explicit-solvent REST2 simulation for compound set C.

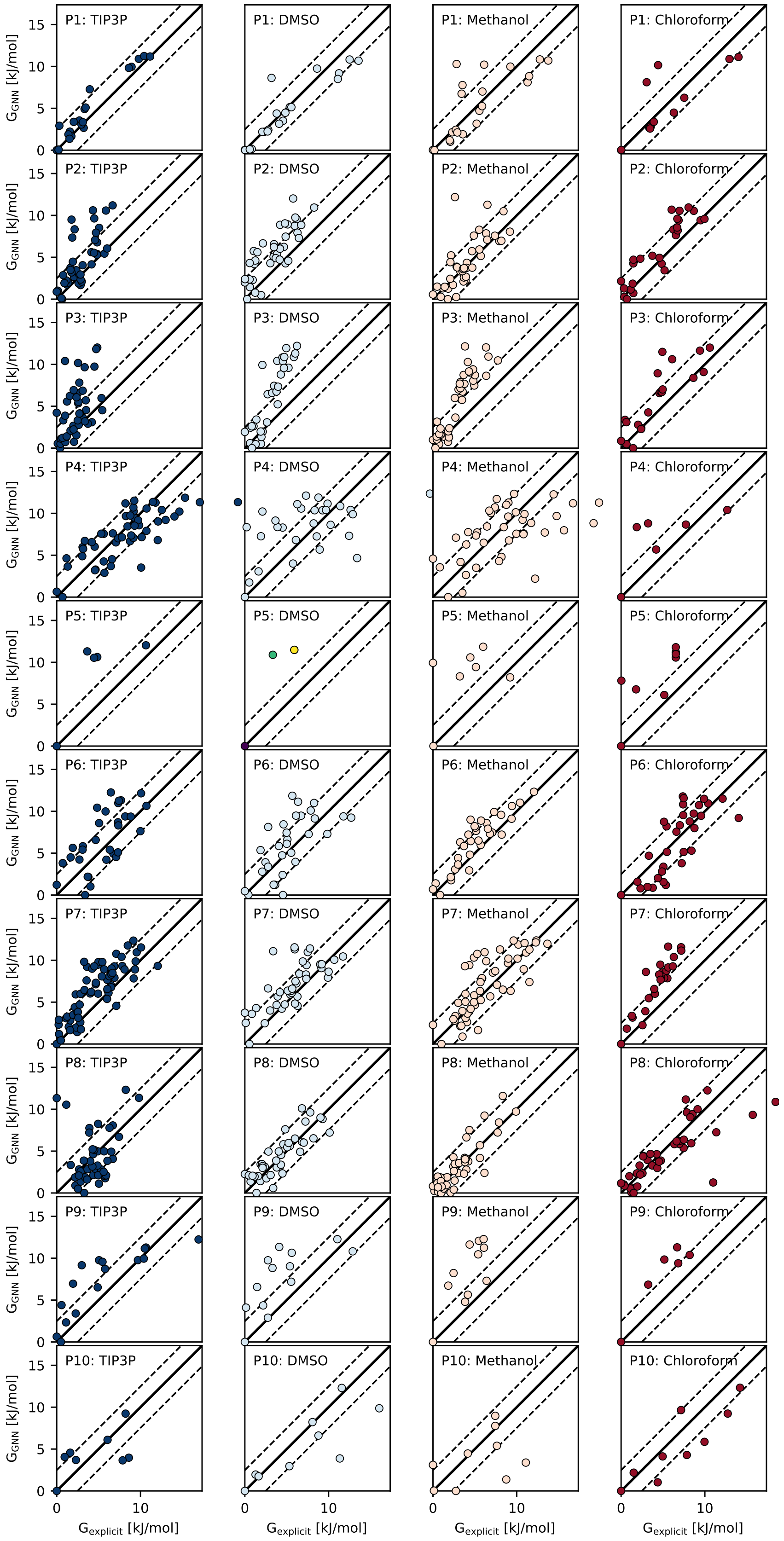

Figure S19: Comparison of the conformational ensemble minimized using the GNNIS model with the explicit-solvent REST2 simulation for compound set P.

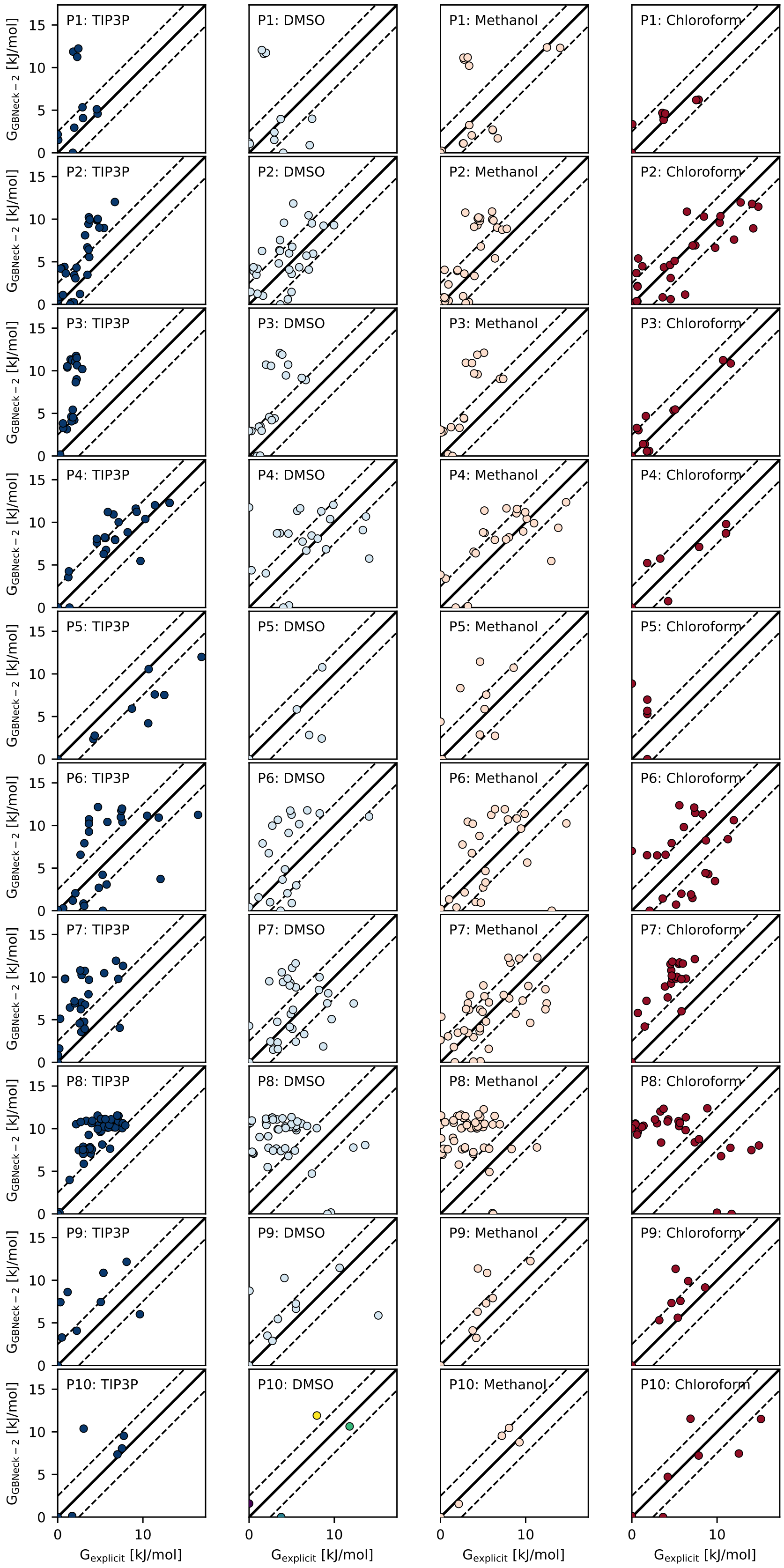

Figure S20: Comparison of the conformational ensemble minimized using the GB-Neck2 implicit solvent model with the explicit-solvent REST2 simulation for compound set P.

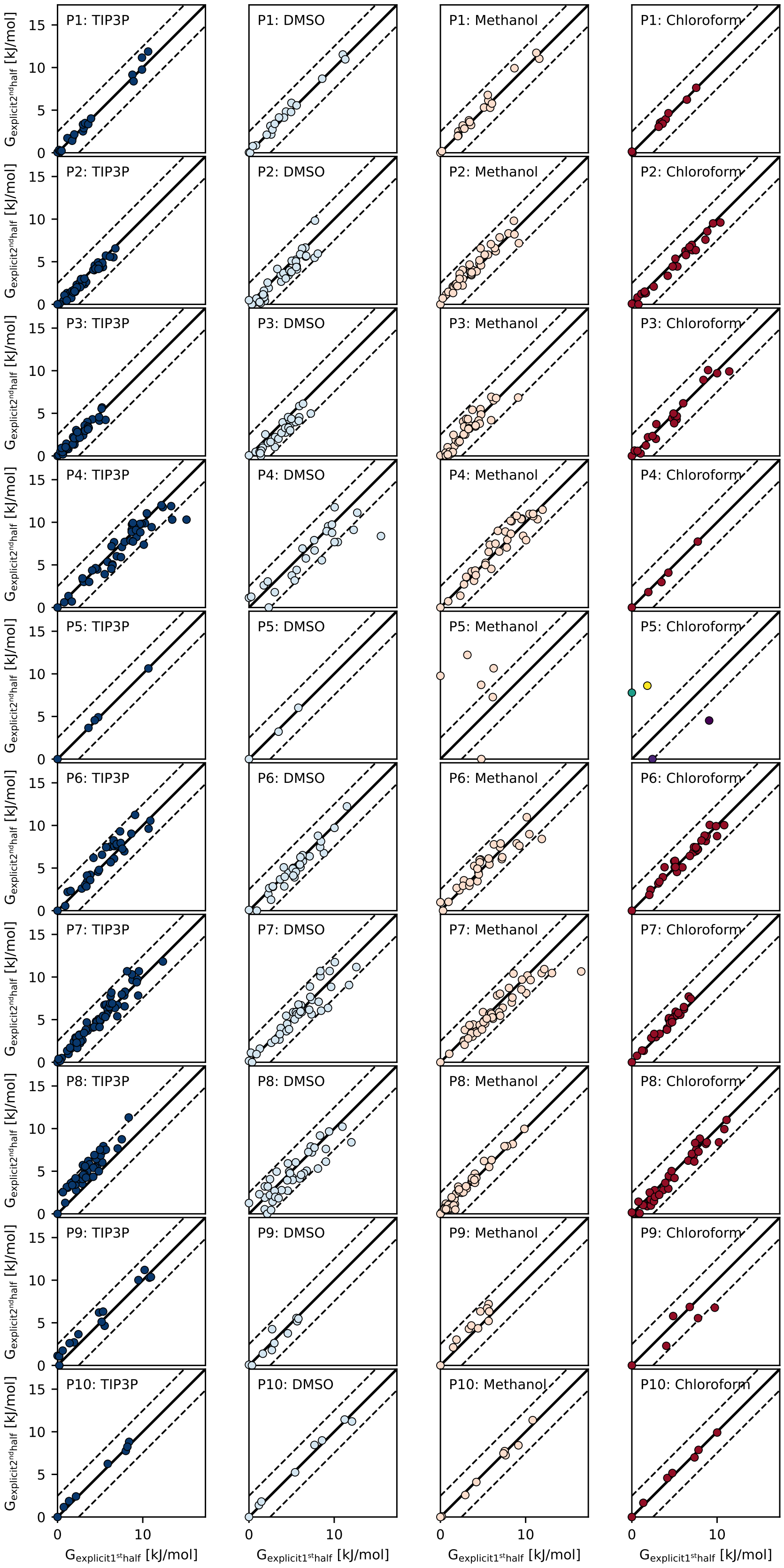

Figure S21: Comparison of the first half of the explicit-solvent REST2 simulations with the second half for compound set P.

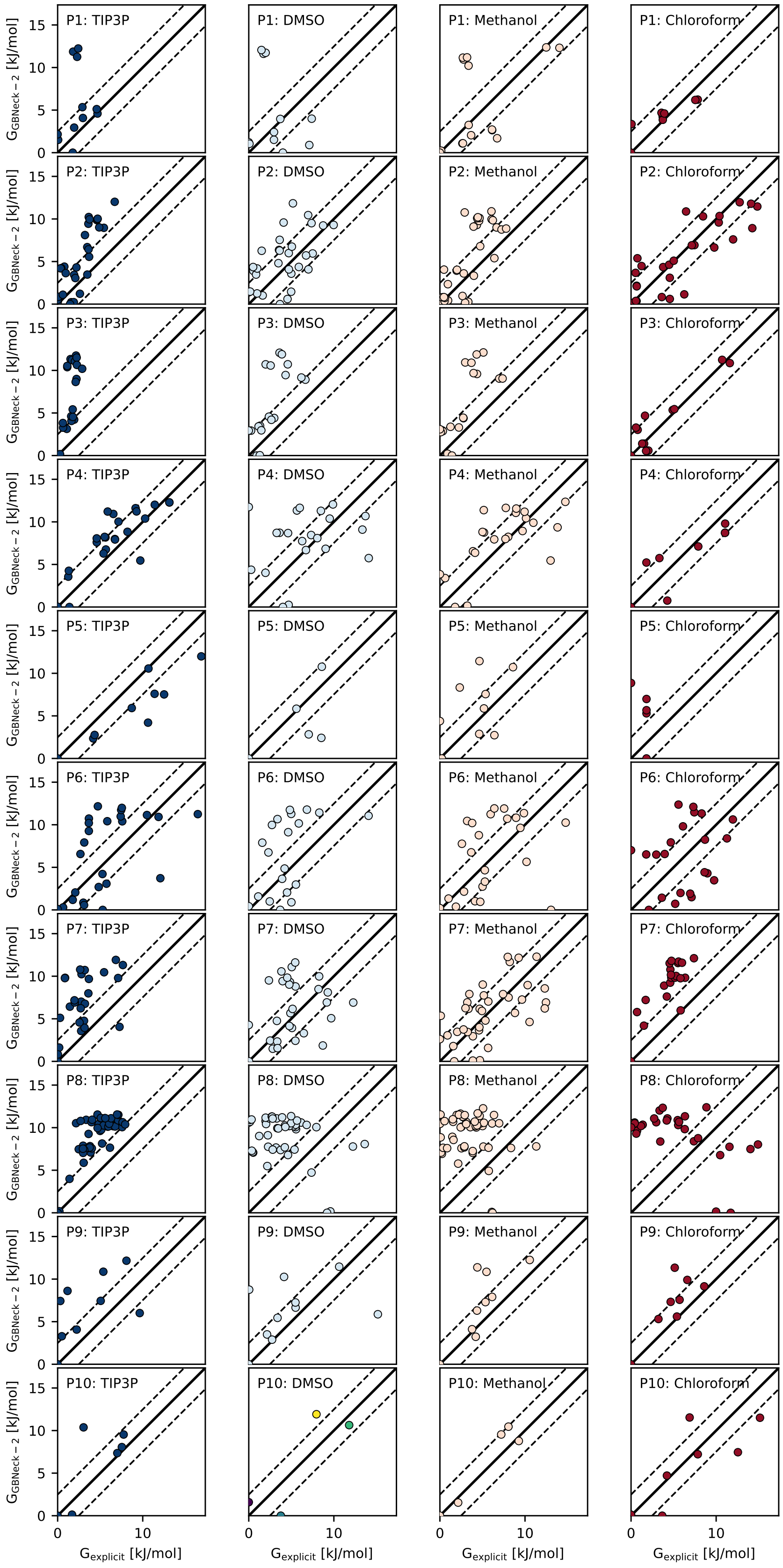

Figure S22: Comparison of the conformational ensemble minimized in vacuum with the explicit-solvent REST2 simulation for compound set P.

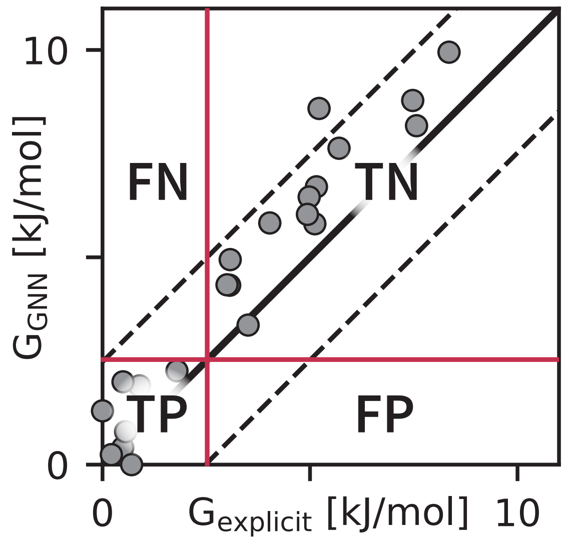

Figure S23: Example of a confusion matrix for compound C1 in water based on a 1 k_BT cutoff.

S8.4 Comparison with Experimental Observables

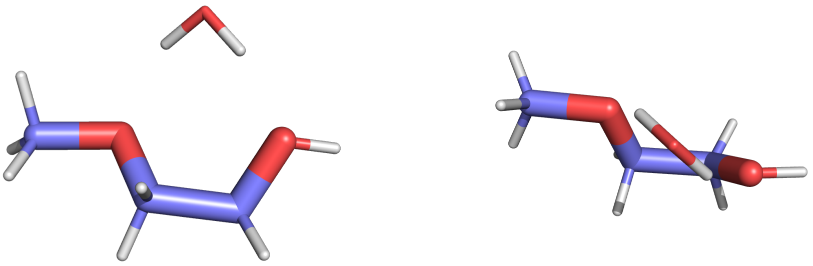

Figure S24: Example of one frame (side view (left) and top view (right)) of the explicit solvent simulation showing a water mediated H-Bond network beliefed to stabilize the gauche conformer.

S8.5 Molecular Balances

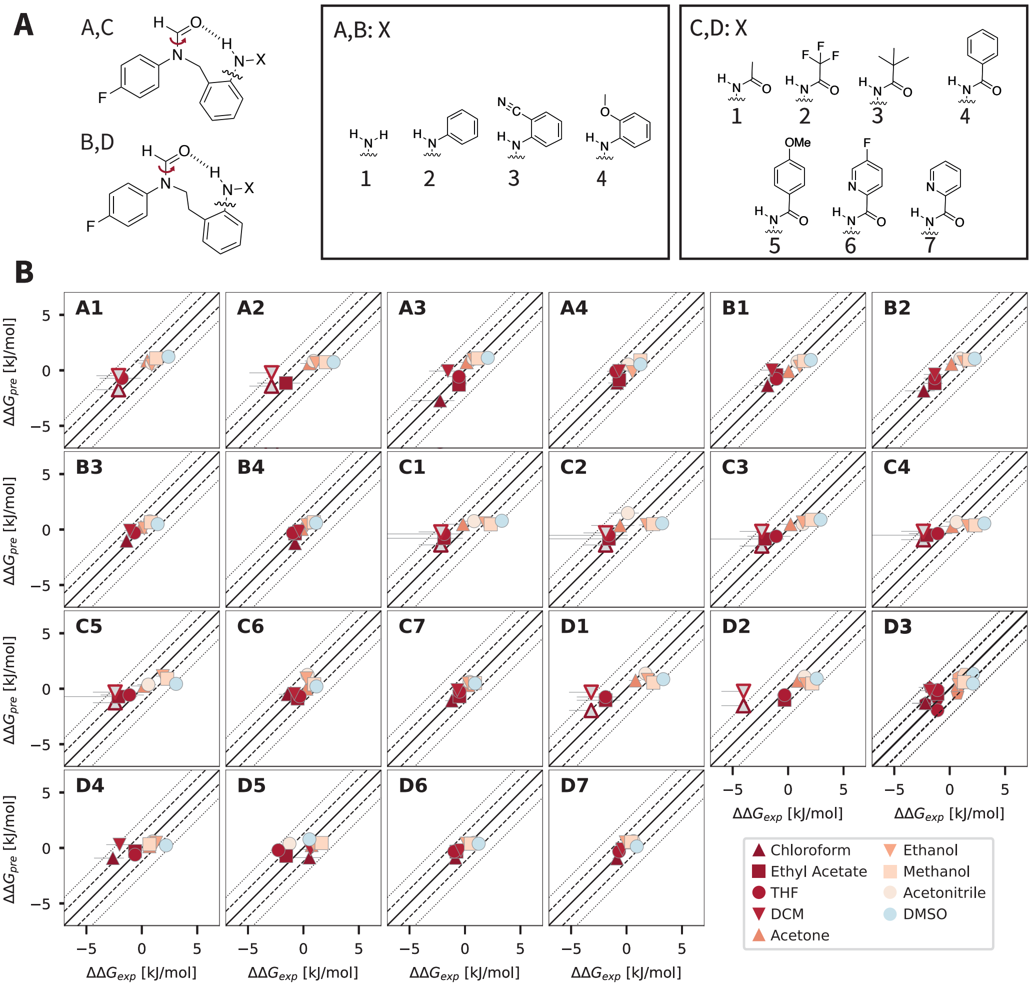

Figure S25: (**A**): Illustration of the 22 studied molecular balances. (**B**): Comparison of predicted $\Delta\Delta G$ values using the GB-Neck2 implicit solvent model and the experimental reference for the 22 molecular balances in nine different solvents. The color scale indicates the dielectric permittivity of the solvent. The experimentally determined standard error is denoted by the black error bars. Dots filled with gray represent measurements where the experiment could not determine the free energy difference between the two rotations exactly. In these cases the value indicated for these points provides an upper limit to the undefined values.

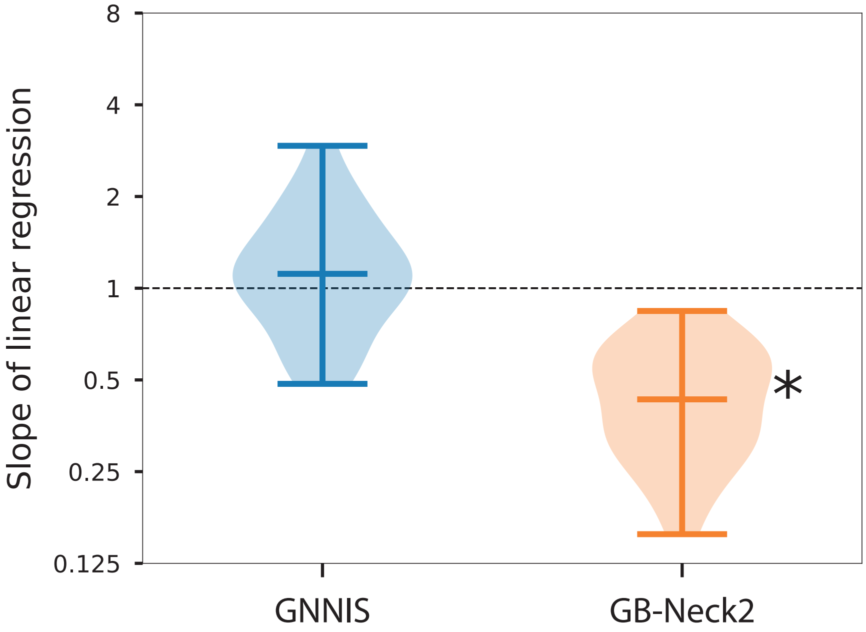

Figure S26: Distribution over linear regression slopes when comparing the implicit-solvent results with the explicit-solvent results for the 22 molecular balances in nine solvents (data shown in Figure 5 in the main text and in Figure S25). The black star indicates a statistically significant difference from the ideal value of 1.

References

[1] Nguyen, H.; Roe, D. R.; Simmerling, C. Improved Generalized Born Solvent Model Parameters for Protein Simulations. *J. Chem. Theory Comput.* **2013**, *9*, 2020–2034.

[2] *CRC Handbook of Chemistry and Physics*; Cleveland, Ohio : CRC Press, c1977-, 2024.

[3] Fujinaga, T.; Izutsu, K.; Sakura, S. Hexamethylphosphoramide: Purification and Tests for Purity. *Pure Appl. Chem.* **1975**, *44*, 115–124.

[4] Rosenfarb, J.; Huffman, H. L. J.; Caruso, J. A. Dielectric constants, viscosities, and related physical properties of several substituted liquid ureas at various temperatures. *J. Chem. Eng. Data* **1976**, *21*, 150–153.

[5] Pérez de la Luz, A.; Iuga, C.; Vivier-Bunge, A. An Effective Force Field to Reproduce the Solubility of MTBE in Water. *Fuel* **2020**, *264*, 116761.

[6] Kirk-Othmer, *Kirk-Othmer Encyclopedia of Chemical Technology*, 4th ed.; John Wiley and Sons, 1995; Vol. 1.

[7] Wohlfahrt, C. 2 Pure Liquids: Data: Datasheet from Landolt-Börnstein - Group IV Physical Chemistry · Volume 6: "Static Dielectric Constants of Pure Liquids and Binary Liquid Mixtures" in SpringerMaterials. `https://materials.springer.com/lb/docs/sm_lbs_978-3-540-47619-1_2`, Copyright 1991 Springer-Verlag Berlin Heidelberg.

[8] Onufriev, A.; Bashford, D.; Case, D. A. Exploring Protein Native States and Large-Scale Conformational Changes with a Modified Generalized Born Model. *Proteins: Struct., Funct., Bioinf.* **2004**, *55*, 383–394.

[9] Katzberger, P.; Riniker, S. A General Graph Neural Network Based Implicit Solvation Model for Organic Molecules in Water. *Chem. Sci.* **2024**, *15*, 10794–10802.

[10] Grimme, S. Supramolecular Binding Thermodynamics by Dispersion-Corrected Density Functional Theory. *Chem. Eur. J.* **2012**, *18*, 9955–9964.
